# Neighborhood Social Disorganization in Early Adolescence and Substance Use Trajectories into Young Adulthood: The Moderating Role of Effortful Control and Parental Substance Use

**DOI:** 10.1007/s10964-025-02270-0

**Published:** 2025-10-08

**Authors:** Yi Zeng, Marco Helbich, Heiko Schmengler, Margot Peeters, Gonneke W. J. M. Stevens

**Affiliations:** 1 Department of Human Geography and Spatial Planning, Utrecht University, Utrecht, the Netherlands; 2 Department of Interdisciplinary Social Science, Utrecht University, Utrecht, the Netherlands

**Keywords:** Neighborhood social disorganization, Substance use, Parental substance use, Effortful control, Adolescence, Young adulthood

## Abstract

Adolescents living in socially disorganized neighborhoods may consume substances more frequently. Although some evidence suggests that such potential neighborhood influences persist into young adulthood, findings remain inconsistent. More research is needed that includes different indicators of neighborhood social disorganization, substance use outcomes, and family- and individual-level moderating factors. To address these gaps, this study examined the associations between three indicators of neighborhood social disorganization—social fragmentation, socioeconomic deprivation, and disorder—in early adolescence and cigarette, alcohol, and cannabis use trajectories into young adulthood in the Netherlands, and the moderating roles of effortful control and parental substance use. Five waves (around age 11–22) from the TRAILS (TRacking Adolescents’ Individual Lives Survey; wave 1: *n* = 2229; age = 11.11 ± 0.56; 50.7% girls) cohort data were used. Higher social fragmentation was associated with trajectories marked by early peaking and heavy increases in alcohol use, and moderate increases in cigarette use. Higher disorder was associated with a trajectory of early but low-frequency cannabis use. Lower socioeconomic deprivation was associated with trajectories showing both moderate and heavy increases in alcohol use. These associations were mostly not moderated by effortful control and parental substance use. These findings suggest that neighborhood social disorganization in early adolescence is associated with distinct substance use trajectories into young adulthood. The patterns vary across both indicators of social disorganization and substances, highlighting the need for targeted, place-based interventions that account for these differences.

## Introduction

Adolescent substance use is a pressing public health concern, as early experimentation often progresses to habitual use and increases the risk of developing substance use disorders and mental health problems later in life (Hall et al., 2016). There is mounting evidence that neighborhood-level factors may contribute to adolescent substance use above and beyond individual- and family-level influences (Jackson et al., 2014; Karriker-Jaffe, 2011). It, however, remains unclear whether and to what extent different indicators of neighborhood social disorganization in early adolescence, when substance use first emerges and values toward it are particularly malleable (Degenhardt et al., 2016), are related to substance use trajectories over the course of development. These potential long-term neighborhood effects may not act in isolation but may interact with family- and individual-level factors (Bronfenbrenner, 1977). To address these gaps, this study (1) assessed the long-term associations between three indicators of neighborhood social disorganization—socioeconomic deprivation, social fragmentation, and disorder—in early adolescence and trajectories of cigarette, alcohol, and cannabis use up until young adulthood; and (2) examined whether effortful control and parental substance use in early adolescence moderate these associations.

### Neighborhood Social Disorganization and Youth Substance Use

Social Disorganization Theory was originally developed to explain why youth deviant behaviors, particularly delinquency, are more common in neighborhoods with disorganized social environments (Shaw & McKay, 1942). Adolescent substance use, particularly in early adolescence, is often conceptualized as a form of deviant behavior (Jessor, 2017). According to this theory, socially disorganized neighborhoods are less able to maintain effective social control over substance use among both adolescents and adults (Sampson & Groves, 1989; Sampson et al., 2002). A lack of positive adult role models regarding substance use may, in turn, further encourage adolescent substance use. Socially disorganized neighborhoods may also be less able to prevent the supply of drugs by dealers and intervene in local drug-selling activities (Logan & Molotch, 2007). This increases drug availability, which may contribute to adolescent substance use. As adolescent substance use is often a group-based behavior, the high prevalence of adolescent substance use could also be due to the inability of socially disorganized neighborhoods to supervise deviant peer groups (Leventhal & Brooks-Gunn, 2000).

Several approaches have been proposed to capture neighborhood social disorganization. Because neighborhoods characterized by concentrated poverty, residential instability, and ethnic heterogeneity may be more socially disorganized (Shaw & McKay, 1942), these structural factors are frequently used as indirect indicators of social disorganization. Neighborhood disorder (e.g., crime), often considered a consequence of social disorganization, is also used as a proxy for this phenomenon (Leventhal & Brooks-Gunn, 2000). Social disorganization can also be assessed more directly through social processes within disorganized neighborhoods, such as a lack of social control and collective efficacy (Sampson et al., 1997).

Studies directly assessing neighborhood social processes in relation to youth substance use are rare (Jackson et al., 2014). Most studies include structural factors (particularly concentrated poverty or socioeconomic deprivation) or neighborhood disorder to assess social disorganization. These studies are predominantly cross-sectional, while longitudinal studies examining substance use trajectories over the course of development—from early adolescence to young adulthood—remain relatively limited and show inconsistent results (Jackson et al., 2014). For example, some studies found that more socioeconomic deprivation during late childhood or early adolescence was associated with higher risks of alcohol use disorders (Karriker-Jaffe et al., 2018) and drug abuse in early adulthood (Sellström et al., 2011), whereas others found no such associations with cigarette use (Mathur et al., 2013), alcohol use (Brenner et al., 2011), and cannabis use (Milliren et al., 2017) in late adolescence or young adulthood. Results are relatively more consistent for neighborhood disorder: exposure to disorder in early adolescence has been linked to more alcohol use (Barr, 2018) and cannabis use in young adulthood (Tarter et al., 2009).

Notwithstanding the knowledge generated from these studies, several limitations remain. First, few studies considered multiple indicators of social disorganization and substance use outcomes. It, therefore, remains unclear whether findings are similar across different indicators of social disorganization and across different types of substance use. This may be an important limitation, as some scholars argued that disorder (e.g., crime) may be a more direct indicator of social disorganization than structural factors (e.g., poverty, residential instability) (Leventhal & Brooks-Gunn, 2000), such that the former may be more strongly associated with youth substance use than the latter. Among structural factors, concentrated poverty and residential instability may be more strongly linked to more youth substance use than ethnic heterogeneity. For example, youth living in neighborhoods with more individuals with immigrant backgrounds may be influenced by religious or cultural norms that discourage alcohol consumption (van Tubergen & Poortman, 2010), resulting in lower levels of alcohol drinking (Fagan et al., 2015). Thus, neighborhood poverty, residential instability, and disorder appear to be more relevant indicators of social disorganization for youth substance use. Associations between neighborhood social disorganization and youth substance use may also differ across substances. Weaker associations for alcohol use than for cigarette and cannabis use may be found, especially in countries where early alcohol use is considered normative (Peeters et al., 2019). More studies incorporating multiple indicators of social disorganization and substance use outcomes may be needed to fill these knowledge gaps.

Second, previous studies are mostly US-based, and it is unclear whether their results are generalizable to other countries. For example, neighborhoods in the US are more residentially segregated than in many other countries (Iceland & Wilkes, 2006). Such strong segregation may strengthen neighborhood effects on youth substance use trajectories in the US context (Karriker-Jaffe, 2011). Adolescents from less segregated countries may also spend more time in different types of neighborhoods, thereby weakening the influence of their residential neighborhoods on their substance use. Such cross-national differences may limit direct extrapolation of US-based findings to other contexts. More effort is therefore needed to investigate relationships between neighborhood social disorganization and youth substance use trajectories outside the US.

### The Moderating Role of Parental Substance Use and Adolescents’ Effortful Control

The associations between neighborhood social disorganization and youth substance use may be moderated by factors at the family and individual levels (Bronfenbrenner, 1977). Parents serve as role models in the family context, and their substance use may amplify the adverse effects of lacking social control and positive adult role models—prevalent in socially disorganized neighborhoods—on youth substance use. Three studies investigated the moderating role of parental substance use in such associations, and their findings generally supported this idea. One study indicated that living in areas with higher crime rates was associated with a higher risk of alcohol initiation only among adolescents who had observed parental drinking (Chen et al., 2016). Another study showed that the positive association between access to substances in the neighborhood and substance use was stronger among adolescents with at least one parent having a history of alcohol or drug abuse (Zimmerman & Farrell, 2017). The third found that only among adolescents with alcohol-addicted parents, greater exposure to neighborhood socioeconomic deprivation was associated with more alcohol use (Trim & Chassin, 2008). Among those with non-addicted parents, greater exposure to socioeconomic deprivation was unexpectedly related to less alcohol use (Trim & Chassin, 2008).

Adolescents’ effortful control (i.e., the self-regulatory capacity to regulate attention and behaviors (Eisenberg et al., 2004)) may act as an individual-level moderator of the association between neighborhood social disorganization and adolescent substance use. Different theoretical explanations suggest that effortful control may either strengthen or weaken this association. The first perspective suggests that adolescents who have more difficulties in regulating their behaviors (e.g., being impulsive and with weaker effortful control) may also have more difficulties in withstanding the neighborhood influence on their substance use (Pluess, 2015). Two studies support this notion. One study, while not specifically focused on impulsivity or effortful control, found that the positive association between perceived neighborhood risk (e.g., drug deals) and substance use was stronger among adolescents higher in sensation seeking—a trait closely linked to impulsivity and characterized by attraction to novel and exciting experiences (Burdzovic Andreas & Watson, 2016). Another reported a stronger positive association between neighborhood risk and cannabis use among adolescents more inclined toward risk-taking (Scheier et al., 2001).

The second theoretical perspective—known as the disadvantage saturation mechanism (Pinchak & Swisher, 2022)—posits that impulsivity or lack of effortful control itself acts as a main risk factor for substance use (Verdejo-García et al., 2008), with neighborhood social disorganization adding little or no additional risk to adolescents who are already highly impulsive and low in effortful control. Two studies support this line of reasoning. One study found a weaker positive association between neighborhood disorder and substance use among adolescents with weaker impulse control (Ray et al., 2016). Another study reported that the positive associations between neighborhood socioeconomic deprivation and cigarette, alcohol, and cannabis use were weaker among adolescents higher in sensation seeking (Jensen et al., 2017).

### Non-random Residential Selection

Residential selection is not random but often shaped by individuals’ preferences, resources, and restrictions (Boschman, 2015). For adolescents, neighborhood contexts are primarily determined by parental choices and constraints (Morris et al., 2018). Adults with immigrant backgrounds, lower socioeconomic status (SES), or mental health problems, as well as families with divorced parents, are more likely to live in socially disorganized neighborhoods (Tunstall et al., 2014; van Ham et al., 2013; van Lenthe et al., 2007). These parental characteristics may also be associated with adolescent substance use (Degenhardt et al., 2016). Adjustment for these selection factors may therefore be needed to mitigate residual confounding bias.

## Current Study

Few studies have assessed long-term associations between neighborhood social disorganization and substance use trajectories during adolescence and young adulthood. It also remains unclear whether these associations vary by indicators of social disorganization, substance use outcomes, or family- and individual-level moderating factors. To fill these gaps, this study assessed whether different indicators of neighborhood social disorganization around age 11 are associated with trajectories of cigarette, alcohol, and cannabis use from around age 14 to 22 in the Netherlands. Neighborhood disorder and two structural factors—socioeconomic deprivation (a broad measure capturing concentrated poverty) and social fragmentation (a broad measure capturing residential instability)—were included to assess social disorganization. Neighborhood ethnic heterogeneity, a commonly used indicator of social disorganization, was not included because adolescents from more ethnically diverse neighborhoods may even have lower substance use, which is inconsistent with Social Disorganization Theory. It was hypothesized that adolescents exposed to more socioeconomic deprivation, social fragmentation, and disorder at age 11 are more likely to follow trajectories of higher cigarette, alcohol, and cannabis use from age 14–22 (Hypothesis 1). As disorder may be a more direct indicator of social disorganization than structural characteristics, disorder was expected to have stronger associations with substance use than the other two. Given the social acceptance of early alcohol use in the Netherlands, its associations with neighborhood social disorganization were expected to be weaker than those of cigarette and cannabis use. This study also assessed the moderating roles of effortful control and parental substance use in these associations. These associations were expected to be stronger among adolescents whose parents consume more substances (Hypothesis 2), and adolescents with weaker (Hypothesis 3) or stronger (Hypothesis 4) effortful control at age 11.

## Methods

### Study Population

This study used longitudinal data from the TRacking Adolescents’ Individual Lives Survey (TRAILS), a prospective cohort study in the northern Netherlands. Of all 135 primary schools in Groningen, Friesland, and Drenthe provinces, 122 schools participated in TRAILS. 2,229 adolescents from these schools, born between 1^st^ October 1989 and 30^th^ September 1991, were included in the baseline survey. Data from the first five survey waves were used, including respondents aged around 11, 13, 16, 19, and 22 years (*T*_*1*_: March 2001-July 2002; *T*_*2*_: September 2003-December 2004; *T*_*3*_: September 2005-August 2007; *T*_*4*_: October 2008-September 2010; *T*_*5*_: March 2012-November 2013). Approximately 80% of participants remained in the study from *T*_*1*_ to *T*_*5*_ (*T*_*2*_: *n* = 2,148; *T*_*3*_: *n* = 1,818; *T*_*4*_: *n* = 1,880; *T*_*5*_: *n* = 1,781). An in-depth description of TRAILS can be found elsewhere (Huisman et al., 2008). All participants provided informed consent before study participation. Ethical approval was obtained from the Dutch Central Committee on Research Involving Human Subjects (#NL38237.042.11).

### Measures

#### Youth Cigarette Use

Cigarette use was assessed from *T*_*2*_ to *T*_*5*_ by asking youth how many cigarettes they smoked per day in the past four weeks. The responses were recoded as follows (Schmengler et al., 2023): 0 (non-smokers), 1 (<1 cigarette per day), 3 (1–5 cigarettes per day), 8 (6–10 cigarettes per day), 15 (11–20 cigarettes per day), and 21 (>20 cigarettes per day). At *T*_*4*_ and *T*_*5*_, more detailed response options expanding ‘>20 cigarettes per day’ to ‘25 (21–30 cigarettes per day)’ and ‘31 (>30 cigarettes per day)’ were included.

#### Youth Alcohol Use

Alcohol use from *T*_*2*_ to *T*_*5*_ was assessed using the quantity-frequency score (McKenna et al., 2018). The frequency score was measured by asking youth how many weekdays (Monday to Thursday) and weekend days (Friday to Sunday) they drank alcohol; the quantity score was assessed based on the self-reported average glasses of alcohol they drank on a regular weekday and weekend day. At *T*_*2*_ and *T*_*3*_, the drinking quantity on a weekday was assessed on a nine-point scale and recoded as follows: 0 (never drink), 1 (1 glass per day) to 6 (6 glasses per day), 8 (7–10 glasses per day), 11 (>10 glasses per day), while responses for weekend days (an 11-point scale) expanded ‘>10 glasses per day’ to 12 (11–14 glasses per day), 17 (15–19 glasses per day), and 20 (20 glasses or more). At *T*_*4*_ and *T*_*5*_, the quantity of use on both weekdays and weekend days was assessed by the same 11-point scale (from ‘never drink’ to ‘20 glasses or more’) as those measured on weekend days at *T*_*2*_ and *T*_*3*_. The frequency scores for weekdays and weekend days were each multiplied by their corresponding quantity score and then summed to obtain the quantity-frequency score.

#### Youth Cannabis Use

Cannabis use from *T*_*2*_ to *T*_*5*_ was assessed by asking youth how many times they consumed cannabis in the past four weeks. The 14-point scale response was recoded to: 0 (never use), 1–10 (ten consecutive categories corresponding to exact usage frequencies), 15 (11–19 times), 29 (20–39 times), and 40 (40 times or more).

#### Neighborhood Social Disorganization

Socioeconomic deprivation, social fragmentation, and disorder at *T*_*1*_ were used as indicators of social disorganization. Four-digit postal code areas were used to delineate the residential neighborhoods. In total, adolescents lived in 113 neighborhoods at *T*_*1*_.

##### Neighborhood socioeconomic deprivation

Socioeconomic deprivation was assessed using administrative register data on all residents living in each neighborhood on January 1^st^, 2005 (Hagedoorn et al., 2020). Z-scores of three sub-indicators—reverse-coded standardized median household income, unemployment rate, and the share of families below the poverty line in each neighborhood—were summed to obtain the deprivation index. Higher scores indicate higher deprivation.

##### Neighborhood social fragmentation

Social fragmentation was measured based on the Congdon index, using the summed z-scores of the proportions of unmarried adults, single-person households, and newly moved-in residents in the previous year in each neighborhood (Congdon, 1996). This measure not only considered residential instability (i.e., newly moved-in residents) but also the share of non-family households (i.e., unmarried adults, single-person households). Such households tend to be more socially isolated and weakly tied to the local community (Congdon, 2004). Data on these indices were from the population-wide registers as of January 1^st^, 2005 (Hagedoorn et al., 2020). Higher scores indicate higher social fragmentation.

##### Neighborhood disorder

Neighborhood disorder was measured using a composite score of safety from Leefbaarometer 1.0 in 2002 (Liveability scores, 2022). The safety index (reverse coded) was constructed based on violent crimes, car thefts, disruption of public order, vandalism, and nuisance in each neighborhood. The score ranges from −50 to 50 (zero indicates the national average). Higher scores indicate higher levels of disorder.

#### Parental Cigarette Use

At *T*_*1*_, each parent was asked about their smoking frequency over the past year, using a six-point scale ranging from ‘never’ to ‘more than two packs a day’. Recoding of the response categories followed the same procedure as that for youth cigarette use (e.g., 5 for ‘1–10 cigarettes a day’). The higher frequency of cigarette use between the mother and father was used for subsequent analysis of youth cigarette use.

#### Parental Alcohol Use

This variable at *T*_*1*_ was measured on a six-point scale, ranging from ‘never’ to ‘more than 20 glasses per week’, based on each parent’s reported alcohol consumption in the past year. The response categories were recoded similarly to those for youth alcohol use (e.g., 15 for ‘11–20 glasses a week’). The higher frequency of consumption between the mother and father was used for subsequent analyses of youth alcohol use.

#### Parental Addiction

Given that data on parental cannabis use is not available at *T*_*1*_, parental addiction was used as the moderator in the analysis for youth cannabis use. Parental addiction was measured by asking (yes or no) if either the mother or father has ever been addicted to one or more substances, including soft drugs (cannabis use), hard drugs, alcohol, sleeping and sedatives, gambling, and others.

#### Adolescents’ Effortful Control

Effortful control around age 11 was assessed using the parent-report Early Adolescent Temperament Questionnaire at *T*_*1*_ (Oldehinkel et al., 2004). To construct effortful control, the mean score of 11 items on a five-point scale (from ‘almost never true’ to ‘almost always true’; Cronbach’s alpha: 0.86) was used. Higher scores indicate stronger effortful control.

#### Sex

Sex was coded as female and male.

#### Ethnicity

Adolescents with at least one parent born in a non-Western country were identified as immigrants. The rest were identified as native (Vollebergh et al., 2005).

#### Family Socioeconomic Status

Family SES at *T*_*1*_ was measured as the average standardized score of both parents’ education, occupation levels, and family income (Ganzeboom & Treiman, 1996). Higher scores indicate higher family SES.

#### Parental Divorce

This variable indicates whether adolescents experienced a divorce (yes or no) between their biological parents before *T*_*1*_.

#### Parental Internalizing Problems

Lifetime parental internalizing problems were assessed at *T*_*1*_ using the Family History Interview, based on reports of lifetime depression and anxiety for both biological parents (Ormel et al., 2005). Higher scores indicate more parental internalizing problems.

#### Parental Externalizing Problems

Lifetime parental externalizing problems, including substance dependence and antisocial behavior, were measured at *T*_*1*_ through the Family History Interview on both biological parents (Ormel et al., 2005). Higher scores indicate more parental externalizing problems.

#### Adolescents’ Educational Levels

According to the educational status at *T*_*2*_, adolescents were classified into four educational tracks: lower vocational and special educational track, intermediate vocational track, higher vocational track, and academic track (Schmengler et al., 2022). While adolescents’ educational levels at *T*_*2*_ may mediate the association between neighborhood social disorganization at *T*_*1*_ and substance use trajectories from *T*_*2*_ to *T*_*5*_, the education level was included as a control to assess the associations through pathways other than mediation by adolescents’ educational levels.

### Statistical Analyses

#### Main Analyses

A bias-adjusted three-step approach (Vermunt, 2010) was used in *Mplus 8.9* (Muthén & Muthén, 1998–2017) to assess the associations between indicators of neighborhood social disorganization around age 11 and trajectories of cigarette, alcohol, and cannabis use from age 14–22. In step one, latent class growth analysis (LCGA) was used to identify classes of growth trajectories for each substance use outcome from *T*_*2*_ to *T*_*5*_. The multilevel structure and predictors were omitted in this step, as its purpose was solely to describe the potential trajectory patterns in the study sample (Hamaker et al., 2020; Vermunt, 2010). In LCGA, zero-inflated Poisson regressions were used to account for the excessive number of zero counts for cigarette and cannabis use. A Poisson regression was used for alcohol use of which the zeros were less frequent. Full information maximum likelihood was used with robust standard errors to handle missingness for each trajectory. Several models with different class numbers and growth factors (i.e., linear only, linear and quadratic slopes) were fitted. The most optimal model was selected based on the best model fit indicated by 1) the Akaike Information Index (AIC), 2) the Bayesian Information Index (BIC), 3) the bootstrapped likelihood ratio test (BLRT), along with the entropy score (>0.8) as well as ensuring the trajectory interpretability based on the theoretical consideration (Peeters et al., 2019) and an acceptable minimal class size (>2% of the total sample (Peeters et al., 2019)). In step two, adolescents were assigned to the most likely trajectory class according to the latent class posterior distribution obtained in step one.

In step three, multilevel multinomial logistic regression models were fitted to assess the associations between neighborhood-level indicators at *T*_*1*_ and the most likely trajectory class for each substance use outcome, with trajectory misclassification accounted for (Asparouhov & Muthén, 2014; Vermunt, 2010). A neighborhood-level random intercept was included to account for the clustering of observations within neighborhoods. A stepwise adjustment strategy was applied: Model 1 included each neighborhood-level indicator separately; Model 2 jointly included all neighborhood-level indicators; Model 3 extends Model 2 by including the cross-level interaction terms for all neighborhood-level indicators with parental substance use and adolescents’ effortful control. Models 1 and 2 were fitted to test Hypothesis 1; Model 3 was used to test Hypotheses 2–4. All models were conditional on all covariates and moderators. Other substance use outcomes were not included as covariates in models for a given outcome because the three outcomes are correlated, and the remaining two may act as mediators of the association between neighborhood social disorganization and the outcome of interest. Controlling for such mediators could introduce over-control bias (Bailey et al., 2024).

Missing values in all variables included in step three were imputed using multiple imputation by chained equations with the *mice* package in R (van Buuren & Groothuis-Oudshoorn, 2011) under the assumption of missing at random (Enders, 2010). Both the aforementioned multilevel structure and the assumed cross-level interactions were accounted for in the imputation model to be consistent with the analytical model (Enders, 2010). The imputation generated 50 sets with 100 between-imputation iterations. Parameter estimates were averaged over the imputed sets using Rubin’s rules (Rubin, 1987).

#### Sensitivity Analyses

Three sensitivity analyses were conducted to test the robustness of the estimated associations. First, Model 3 was refitted by adding the cross-level interaction terms into Model 1 instead of Model 2, given the moderate to strong correlations among neighborhood-level indicators (Table S1). Second, Models 1 and 2 were refitted by removing parental substance use and adolescents’ effortful control, as both factors may mediate associations between neighborhood social disorganization and youth substance use (Chuang et al., 2005; Esposito et al., 2017). Third, Models 1 and 2 were refitted by replacing the educational level at age 14 (*T*_*2*_) with the one measured at age 19 (*T*_*4*_). This analysis aims to control for the potential unexpected pathway whereby adolescents from less socioeconomically deprived neighborhoods may have higher educational attainment (Nieuwenhuis et al., 2021), and in turn, those with higher education may drink more alcohol during late adolescence and young adulthood (Schmengler et al., 2022).

## Results

### Descriptive Statistics

Table 1 summarizes the sample characteristics. Of the 2,229 participants, 49.3% were boys. Most participants were native (89.4%), had not experienced parental divorce (78.6%), and had parents without a history of addiction to substances (91.9%). Participants were evenly distributed across the four educational tracks, though a slightly higher proportion was in the lower vocational track (32.2%).

**Table 1:** Descriptive Statistics of the Study Population

	Proportion or mean (standard deviation)				
Variable	Wave 1	Wave 2	Wave 3	Wave 4	Wave 5
	*n* = 2,229	*n* = 2,148	*n* = 1,818	*n* = 1,880	*n* = 1,781
Youth cigarette use		0.70 (3.12)	2.37 (5.22)	3.53 (6.62)	3.63 (6.66)
Youth alcohol use		1.63 (4.48)	6.81 (9.36)	9.98 (11.40)	9.98 (10.71)
Youth cannabis use		0.13 (1.37)	1.17 (5.19)	1.87 (6.65)	1.77 (6.45)
Age	11.11 (0.56)	13.57 (0.53)	16.28 (0.71)	19.08 (0.60)	22.29 (0.65)
Sex: Male	49.3				
Ethnicity: Immigrant	10.6				
Parental divorce: Yes	21.4				
Family socioeconomic status	−0.05 (0.80)				
Parental internalizing problems	0.55 (0.79)				
Parental externalizing problems	0.14 (0.42)				
Adolescents’ effortful control	3.23 (0.68)				
Parental cigarette use	6.31 (8.07)				
Parental alcohol use	5.78 (5.51)				
Parental addiction	8.1				
Adolescents’ educational levels					
Lower vocational & special education		32.2			
Intermediate vocational		25.2			
Higher vocational		19.4			
Academic		23.2			
Neighborhood socioeconomic deprivation	2.22 (2.55)				
Neighborhood social fragmentation	1.68 (2.72)				
Neighborhood disorder (range: -50-50)	9.12 (22.79)				

On average, adolescents’ cigarette use was low around age 14 and increased steadily until about age 22. A similar growth pattern was observed for adolescents’ alcohol and cannabis use from around age 14–19; however, alcohol use stabilized, and cannabis use slightly decreased from around age 19–22.

### Correlations of Neighborhood-level Indicators with Moderators and Covariates

Table S1 presents the pairwise *Spearman* correlations (*r*) among the variables. Moderate to strong correlations (Bosco et al., 2015) among neighborhood socioeconomic deprivation, social fragmentation, and disorder (*r* = 0.58–0.72) were observed. Adolescents living in more socioeconomically deprived, socially fragmented, and disordered neighborhoods were more likely to have an immigrant background (*r* = 0.18–0.22), lower family SES (*r* = −0.07 – –0.32), and divorced parents (*r* = 0.16–0.18). Adolescents’ educational level was correlated only with neighborhood socioeconomic deprivation (*r* = –0.20) and disorder (*r* = –0.08), but not social fragmentation.

Parental characteristics were also correlated with the neighborhood-level indicators. Parents with more externalizing problems (*r* = 0.12–0.16), higher levels of cigarette use (*r* = 0.09 – 0.14), and a history of addiction to substances (*r* = 0.09–0.14) were more likely to live in more socioeconomically deprived, socially fragmented, and disordered neighborhoods. Parental internalizing problems were correlated only with neighborhood socioeconomic deprivation (*r* = 0.08) and disorder (*r* = 0.06), but not social fragmentation. Parental alcohol use was only correlated with socioeconomic deprivation (*r* = –0.12), but in an opposite direction (negative) from correlations of both parental cigarette use and addiction with socioeconomic deprivation (positive).

### Growth Trajectories of Substance Use Outcomes

The LCGA model with the optimal number of classes and growth factors for each substance use outcome based on the model-fit indices, the entropy score, the smallest group size (Tables S2–S4), and trajectory interpretability was selected. The results are consistent with previous research on the TRAILS cohort (Peeters et al., 2019).

#### Cigarette Use

**Fig. 1:**
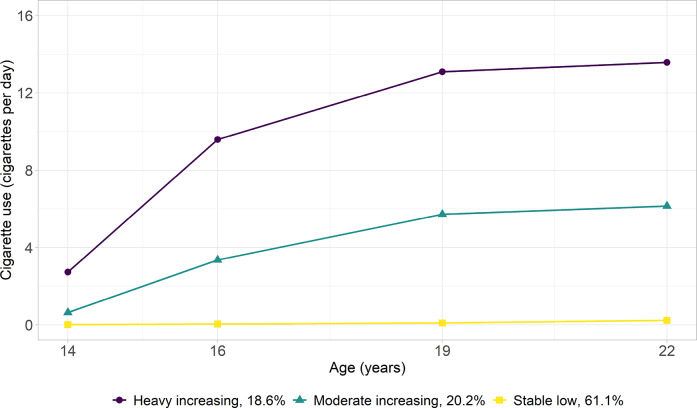
Trajectories of Cigarette Use (Cigarettes Per Day) from Age 14–22 Years. The proportion (%) indicates the percentage of participants assigned to each trajectory

The three-class LCGA model incorporating both linear and quadratic slopes was identified as optimal for cigarette use. Figure 1 shows that most adolescents had consistently low cigarette use from age 14 to 22 (stable low: 61.1%); the remaining adolescents were almost evenly grouped into two other trajectories—moderate increasing (20.2%) and heavy increasing (18.6%)—both showing continuous increases but at different rates.

#### Alcohol Use

**Fig. 2:**
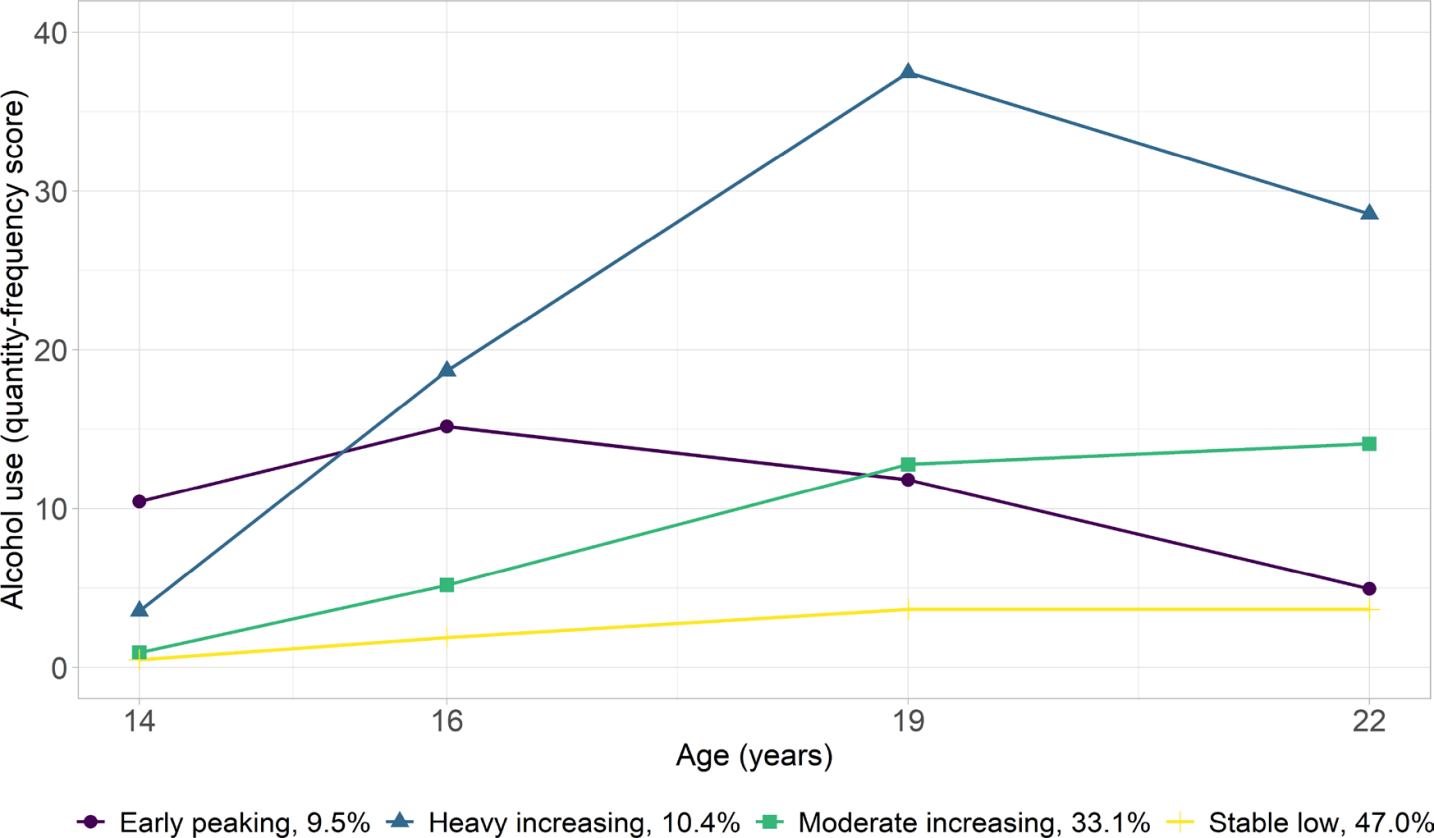
Trajectories of Alcohol Use (Quantity-frequency Score) from Age 14–22 Years. The proportion (%) indicates the percentage of participants assigned to each trajectory

Four classes of trajectories were identified as optimal for alcohol use from the LCGA models combining linear and quadratic slopes (Fig. 2). Two trajectories—stable low (47.0%) and moderate increasing (33.1%)—encompassing most adolescents, showed sustained but small increases in alcohol use from age 14–22. A steeper increase was observed in the heavy-increasing (10.4%) trajectory from age 14–19, followed by a sharp decline until age 22. Adolescents in the early-peaking trajectory (9.5%) typically consumed more alcohol at age 14 than adolescents in other trajectories, but their consumption decreased from age 16 onward.

#### Cannabis Use

**Fig. 3:**
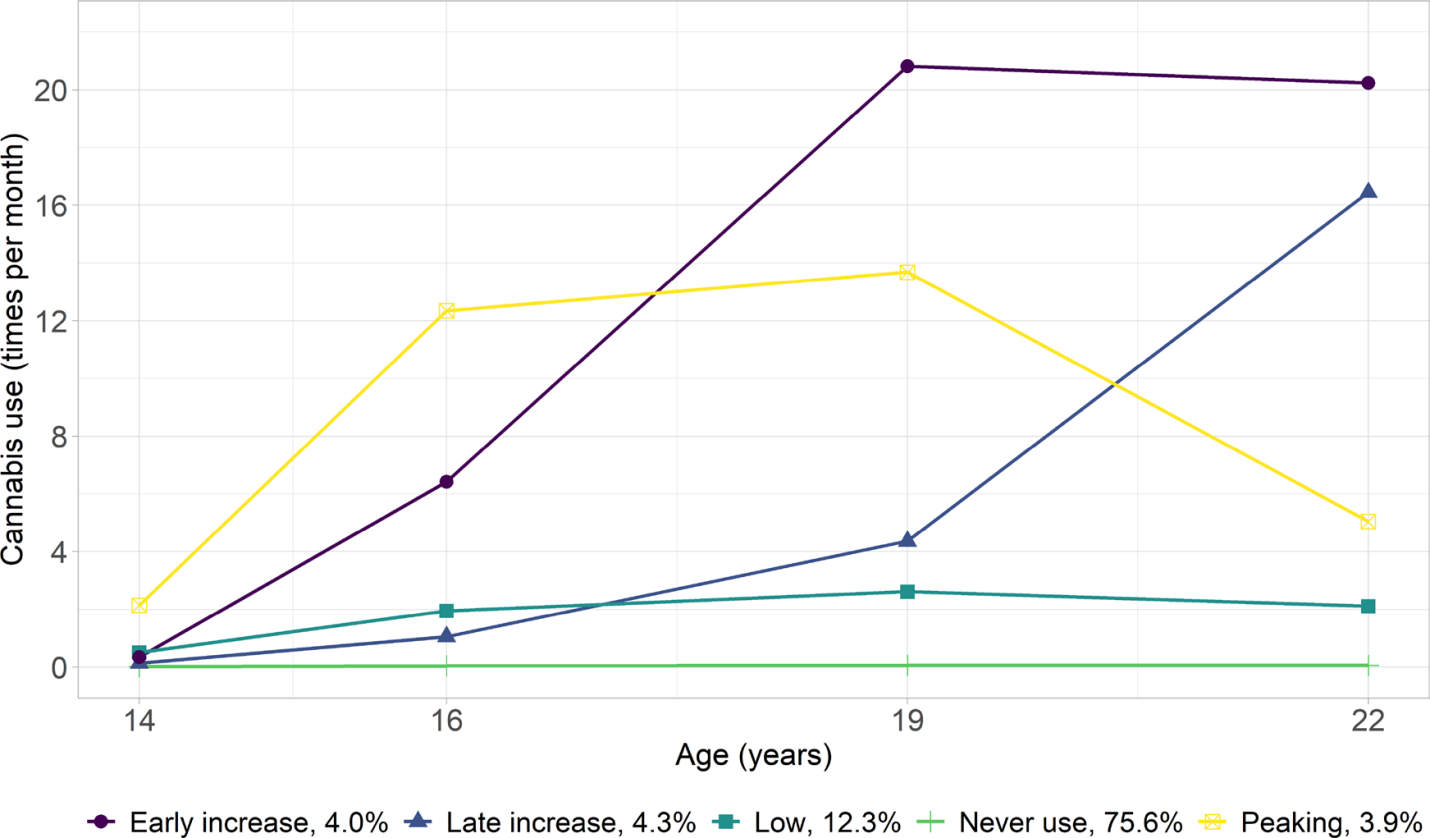
Trajectories of Cannabis Use (Times Per Month) from Age 14–22 Years. The proportion (%) indicates the percentage of participants assigned to each trajectory

The five-class linear-quadratic-slopes LCGA model was found as optimal for cannabis use (Fig. 3). Most adolescents never used cannabis (never use: 75.6%) or rarely used it (low: 12.3%) from age 14 to 22. Two trajectories indicated early patterns of increased cannabis use: one demonstrated a rise from age 14 to 19 and plateaued thereafter (early increase: 4.0%), while the other peaked at 16, remained relatively stable until 19, and declined thereafter (peaking: 3.9%). The other adolescents showed a striking increase in cannabis use starting at age 19, having previously remained at relatively low levels (late increase: 4.3%).

### Associations of Neighborhood-level Indicators with Trajectory Membership for Cigarette, Alcohol, and Cannabis Use

**Fig. 4:**
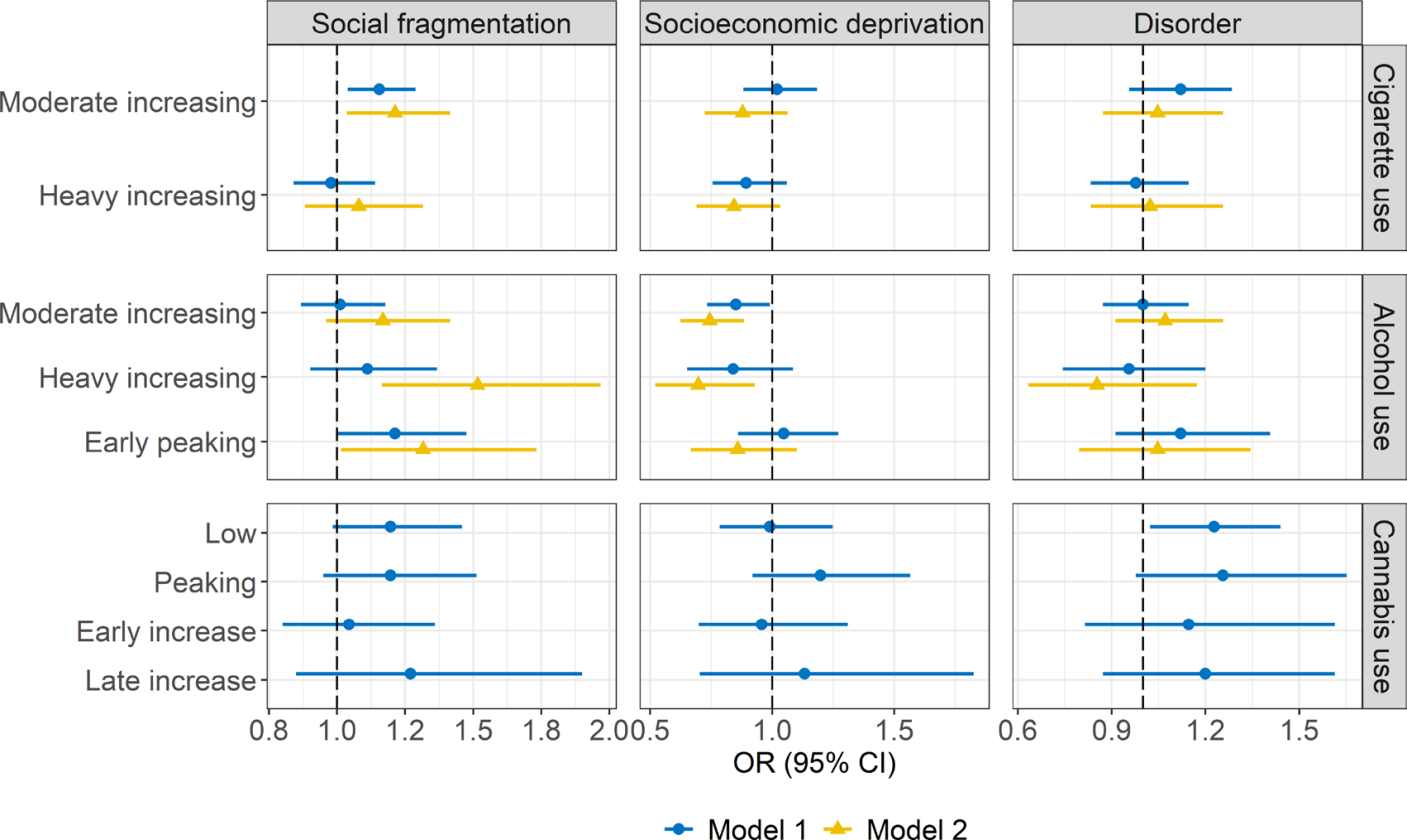
Associations of Neighborhood Social Fragmentation, Socioeconomic Deprivation, and Disorder with Membership in Trajectories of Cigarette, Alcohol, and Cannabis Use. All associations were rescaled by the standard deviation of the corresponding neighborhood-level indicator. The reference trajectory group for cigarette use was ‘stable low’; for alcohol use was ‘stable low’, and for cannabis use was ‘never use’. OR odds ratio; 95% CI 95% confidence interval

Figure 4 and Tables S5–S7 present results of the multilevel multinomial logistic regression models for cigarette, alcohol, and cannabis use.

#### Cigarette Use

Adolescents exposed to higher neighborhood social fragmentation at age 11 were more likely to be in the moderate-increasing trajectory (vs. stable low; Models 1 and 2). The associations for socioeconomic deprivation and disorder were non-significant.

Higher parental cigarette use was associated with higher odds of being in both the moderate- and heavy-increasing trajectories (vs. stable low). Higher effortful control was associated with lower odds of following the moderate-increasing trajectory (vs. stable low)^1^. These two variables did not moderate associations between neighborhood-level indicators and cigarette use trajectories.

#### Alcohol Use

Adolescents exposed to more neighborhood social fragmentation at age 11 were more likely to be in the early-peaking and heavy-increasing trajectories (vs. stable low; only in Model 2). In contrast, adolescents exposed to more neighborhood socioeconomic deprivation at age 11 were less likely to be in the moderate-increasing (Models 1 and 2) and heavy-increasing (only in Model 2) trajectories (vs. stable low). The associations for neighborhood disorder were non-significant.

Higher parental alcohol use was associated with higher odds of following the early-peaking, heavy-increasing, and moderate-increasing trajectories (vs. stable low). Parental alcohol use, however, did not moderate the associations between neighborhood-level indicators and alcohol use trajectories. Effortful control was not associated with any alcohol use trajectory.^1^ This variable, however, moderated the association between neighborhood disorder and the moderate-increasing trajectory (vs. stable low). As shown in a Johnson-Neyman plot (Lazar et al., 2013), such an association was positive among adolescents with effortful control above the average, but negative among those with effortful control below the average (Fig. S1).

#### Cannabis Use

Results for cannabis use showed wider confidence intervals than those for cigarette and alcohol use. These less precise estimates were possibly due to smaller group sizes of some cannabis use trajectories.

Non-significant associations were found for both socioeconomic deprivation and social fragmentation. As for neighborhood disorder, Model 1 showed that adolescents living in more disordered neighborhoods at age 11 were more likely to be in the low trajectory (vs. never use). Model 2 was not fitted for this outcome, as in Model 2, the point estimate of the association for neighborhood disorder remained unchanged from Model 1, but the confidence interval widened substantially. This decreased precision was likely due to limited outcome variance and moderate to strong correlations among neighborhood-level indicators. Thus, in Model 3, the cross-level interaction terms were included in each Model 1, instead of in Model 2 as planned.

Although parental addiction was not associated with any trajectory (vs. never use), among adolescents with at least one parent having a history of substance addiction, more social fragmentation was associated with higher odds of being in the low trajectory (vs. never use). The association was non-significant among those whose parents had not been addicted to substances (Fig. S2). Higher effortful control was related to lower odds of being in the peaking trajectory (vs. never use). This variable did not moderate the associations between neighborhood-level indicators and cannabis use trajectories.

### Post-hoc Analyses

Three associations for alcohol use (i.e., social fragmentation-heavy increasing, social fragmentation-early peaking, and socioeconomic deprivation-heavy increasing (vs. stable low)) were significant only in the model with all three neighborhood-level indicators mutually adjusted (Model 2). This result pattern might be driven by a potential suppression effect (MacKinnon et al., 2000) of the two positively correlated indicators—social fragmentation and socioeconomic deprivation—that were associated with membership in the corresponding trajectory (vs. stable low) in opposite directions. As these associations showed decreased precision (wider confidence intervals than those in Model 1), two post-hoc analyses were conducted to assess their robustness: (1) examined the observation distributions in tabulations to assess the extent of overlap between social fragmentation, socioeconomic deprivation, and disorder (Messer et al., 2010); (2) fitted a simpler model than Model 2 by mutually adjusting for socioeconomic deprivation and social fragmentation, but not disorder. As shown in Supplementary Materials S1, both analyses supported the robustness of these associations.

### Sensitivity Analyses

All estimated associations were robust to the three sensitivity analyses, modifying the model structure by adding interaction terms in Model 1 (rather than in Model 2; Tables S8, S9), excluding potential mediators (parental substance use and adolescents’ effortful control; Table S10–12), and replacing the education variable at age 14 with the one at age 19 (Table S13).

## Discussion

Adolescents living in socially disorganized neighborhoods may consume more substances. Some studies examined whether such associations persist into young adulthood, but their results remain inconsistent. It also remains unclear whether these long-term associations depend on the specific indicators of social disorganization, substance use outcomes, and family- and individual-level moderators. The present study addressed these gaps by investigating the relationships between three indicators of neighborhood social disorganization—socioeconomic deprivation, social fragmentation, and disorder—in early adolescence and developmental trajectories of cigarette, alcohol, and cannabis use into young adulthood, and the moderating roles of parental substance use and adolescents’ effortful control.

It was hypothesized that adolescents living in more socioeconomically deprived, socially fragmented, and disordered neighborhoods in early adolescence are more likely to follow trajectories of higher cigarette, alcohol, and cannabis use into young adulthood. The results only partially aligned with this hypothesis, as the three indicators of social disorganization were not uniformly associated with higher-use trajectories across all substances. It was also expected that disorder would have stronger associations with youth substance use than socioeconomic deprivation and social fragmentation. This expectation was not confirmed, as disorder was not associated with more substance use outcomes than the other two indicators of social disorganization. Alcohol use was expected to have weaker associations with neighborhood social disorganization than cigarette and cannabis use. This was not supported by the findings either, as alcohol use was associated with two out of three indicators of social disorganization, while cigarette and cannabis use were associated with one.

Adolescents from more disordered neighborhoods at age 11 were more likely to initiate cannabis use early (around 16) and maintain low but persistent use until age 22 (the low trajectory vs. the never-use trajectory). Although the frequency of cannabis use in this trajectory was relatively low, early initiation remains a concern, as it is often related to increased risks of substance use disorders and mental health problems later in life (Hall et al., 2016). Disorder was the only neighborhood-level indicator associated with cannabis use, while it showed no association with cigarette or alcohol use. A possible explanation for this is that cannabis use is often perceived as a more deviant behavior than cigarette or alcohol use, and disorder, compared with social fragmentation and socioeconomic deprivation, more directly captures exposure to neighborhood environments where deviant and illegal activities are prevalent.

Neighborhood socioeconomic deprivation was associated only with alcohol use trajectories, but in a direction contrary to the expectation (Hypothesis 1). Adolescents living in less socioeconomically deprived (i.e., more affluent) neighborhoods at age 11 were more likely to follow both the moderate and heavy-increasing alcohol use trajectories (vs. the stable-low trajectory) from age 14 to 22. Other studies reported similar findings. For example, a US study found that adolescents from more affluent neighborhoods had a more rapid increase in alcohol use throughout adolescence (Barr, 2018). More alcohol consumption was also found among adolescents residing in more affluent areas in Norway (Pedersen et al., 2015). One potential explanation for this finding is that more affluent neighborhoods tend to have stronger social cohesion and more frequent social activities with alcohol involved, which may foster positive norms around alcohol use that may influence adolescents’ drinking behaviors (Pedersen et al., 2015). This explanation may particularly be relevant in the Dutch context, where alcohol use is normative and more frequent in more affluent neighborhoods (Kuipers et al., 2013). It could also be that adolescents living in more affluent neighborhoods are more likely to attain higher levels of education (Nieuwenhuis et al., 2021), and higher-educated youth tend to drink more alcohol during the transition to young adulthood (Schmengler et al., 2022). Results from a sensitivity analysis suggest that this educational mediation pathway, however, seems unlikely. Socioeconomic deprivation was not associated with either cigarette or cannabis use. A possible explanation is that, although more affluent neighborhoods may have stronger social cohesion, they are not necessarily better organized to regulate adolescents’ cigarette and cannabis use than less affluent ones, as higher-SES adults may not be actively engaged in local organization and community management due to more work obligations (Kang, 2011).

Social fragmentation was associated with both cigarette and alcohol use trajectories. Adolescents living in more socially fragmented neighborhoods at age 11 were more likely to follow trajectories characterized by moderate increases (vs. the stable-low trajectory) in cigarette use, as well as heavy-increasing and early-peaking alcohol use (vs. the stable-low trajectory) from age 14 to 22. In accordance with Social Disorganization Theory, these results suggest that social fragmentation may lead to a lack of social control and positive adult role models regarding substance use, contributing to increased cigarette and alcohol use (Leventhal & Brooks-Gunn, 2000). Social fragmentation was associated with more substance use trajectories than the other two neighborhood-level indicators. One potential explanation is that social fragmentation may serve as a more comprehensive indicator of social disorganization, as it may more directly reflect resident characteristics—residential instability (newly moved-in residents) and non-family households (single-person households, unmarried adults)—that may contribute to disorganized social environments. These groups are often less embedded in the local community and less likely to participate in neighborhood organizations that regulate adolescent cigarette and alcohol use (Congdon, 2004). An alternative explanation may be that social fragmentation is correlated with other contextual factors (e.g., urbanization, concentration of young adults; (Zeng et al., 2023)) associated with youth substance use. For example, adolescents living in more urbanized areas may smoke and drink alcohol more frequently due to the proximity to bars, clubs, and other retail outlets (Bryden et al., 2012; Van Deelen et al., 2022).

Overall, indicators of social disorganization were mainly associated with substance use trajectories characterized by moderate increases—particularly cigarette and cannabis use—rather than those characterized by heavier escalation. This result contrasts with prior findings, predominantly from the US studies, which largely linked indicators of neighborhood social disorganization in early adolescence to outcomes associated with heavier use—such as nicotine dependence and cannabis use disorders—in early (Tarter et al., 2009) or mid-adulthood (Lee et al., 2017). Such discrepancies are consistent with the notion that neighborhood influences on youth substance use may be stronger in more residentially segregated countries, such as the US (Karriker-Jaffe, 2011). The lack of associations with higher-risk trajectories may also be due to limited statistical power for the smaller-sized trajectories. This is especially relevant for cannabis use, where most associations aligned with expectations but had wide confidence intervals, rendering them non-significant.

It is important to note that the present study tracked adolescents’ substance use over a long-term period, a factor that must be considered when comparing findings to those of prior studies, which are mostly cross-sectional or had shorter follow-up periods. Nearly all substance use trajectories associated with neighborhood social disorganization exhibited low consumption in early and mid-adolescence, followed by more visible increases over time. This pattern implies that the associations between neighborhood social disorganization in early adolescence and substance use may not be immediately evident but instead manifest more apparently as adolescents grow older. One potential explanation is that early to mid-adolescence may still be too young for substance use for many adolescents, but exposure to neighborhood social disorganization could still influence adolescents’ values toward substances, which, in turn, contributes to their later-life substance use. This may help explain, at least partially, why some cross-sectional studies found no association between neighborhood social disorganization and substance use among young adolescents (Jackson et al., 2014).

Contrary to expectations, associations between neighborhood social disorganization and substance use were largely not moderated by parental substance use or adolescents’ effortful control. This may suggest that neighborhood social disorganization may contribute to youth substance use independent of these family- and individual-level influences. This interpretation should, however, be made with caution, as some interaction effects may be underpowered, particularly for substance use trajectories with small group sizes. This speculation may be supported by the two significant interactions observed for larger-size trajectories (i.e., the low cannabis use and the moderate-increasing alcohol use trajectory). In line with Hypothesis 2, higher neighborhood social fragmentation in early adolescence was associated with a higher likelihood of following the low cannabis use trajectory (vs. the never-use trajectory) only among adolescents whose parent(s) had a history of substance addiction. This finding echoes those from a US study, indicating a stronger association between living in neighborhoods with higher accessibility to substances and more substance use among adolescents whose parent(s) had a history of alcohol and/or drug abuse (Zimmerman & Farrell, 2017). A possible explanation is that parents with a history of addiction might still be actively struggling with addiction. Adolescents from such households living in more socially fragmented neighborhoods may lack positive role models for substance use both in the home and neighborhood contexts, which might show combined effects on shaping adolescent cannabis use behaviors.

Effortful control moderated the association between neighborhood disorder and membership in the moderate-increasing alcohol use trajectory (vs. the stable-low trajectory). Among those with effortful control higher than the average, more neighborhood disorder was associated with a higher likelihood of following the moderate-increasing alcohol use trajectory (vs. the stable-low trajectory). This finding partially aligns with Hypothesis 4 and previous research (Jensen et al., 2017; Ray et al., 2016), implying that even adolescents with strong effortful control may exhibit increased alcohol use when exposed to neighborhood disorder. It is important to note that this increased use remained moderate and emerged only in late adolescence—a pattern that may reflect normative consumption rather than high-risk drinking. This may be because while neighborhood disorder may shape adolescents’ drinking behaviors, effortful control may still serve as a protective factor, preventing the escalation into more problematic alcohol use trajectories. In contrast, among adolescents with effortful control below the average, higher neighborhood disorder was related to a lower likelihood of developing the moderate-increasing alcohol use trajectory (vs. the stable-low trajectory). A possible explanation is that parents of adolescents with weak effortful control are more vigilant and thus more inclined to adopt strict parenting strategies to shield their children from the adverse neighborhood effects on alcohol use when living in disordered neighborhoods (Chuang et al., 2005).

A key strength of this study is the use of longitudinal data to identify substance use trajectories from early adolescence to young adulthood. Second, multiple indicators of neighborhood social disorganization alongside diverse substance use outcomes were considered. This comprehensive approach revealed differential associations across indicators of social disorganization and substance use outcomes. Third, this study adds to the literature by exploring the multilevel interactions between factors at the neighborhood-, family-, and individual-level in associations with substance use over time.

There are also some limitations. First, this study included exposure to neighborhood social disorganization at only age 11, without considering the neighborhood-level exposure occurring thereafter. This decision is mainly driven by strong correlations between neighborhood-level exposures across adjacent time points, especially during adolescence. This study was thus unable to differentiate the independent association between each time-specific exposure to neighborhood social disorganization and substance use. The strong correlations among neighborhood-level exposures over time are common in adolescence, when individuals are largely residentially immobile and consistently exposed to similar neighborhood environments (Jackson & Mare, 2007). This issue may be mitigated by focusing on a larger study sample with more adolescents relocating over time. Second, LCGA was used to identify substance use trajectories without modeling the variance in growth factors per trajectory. While this within-class variance can be modeled using a Growth Mixture Model (GMM), such a model, including count variables with an excessive number of zeros, did not converge. LCGA and GMM, nevertheless, often yield similar trajectory outputs (Sijbrandij et al., 2019). Third, some substance use trajectories have small group sizes. Some associations—as well as the interaction terms—with the small-sized trajectories may be underpowered. Fourth, postal code areas were used to approximate residential neighborhoods. These geographic units are, however, not designed to reflect neighborhood boundaries accurately, and the measures of neighborhood social disorganization may therefore be biased (Helbich et al., 2024). Whether this is a real concern is still under debate, as postal codes or other administrative units are predominantly used (Jackson et al., 2014), and some studies showed that the estimated neighborhood effects are robust across units with different spatial granularities (Wheaton & Clarke, 2003).

## Conclusion

Evidence on how neighborhood social disorganization is associated with youth substance use over the course of development remains inconsistent. This association may be dependent on indicators of social disorganization, substance use outcomes, and moderated by individual- and family-level factors. This study examined long-term associations between three indicators of neighborhood social disorganization—socioeconomic deprivation, social fragmentation, and disorder—in early adolescence and cigarette, alcohol, and cannabis use trajectories into young adulthood in the Netherlands, as well as the moderating roles of effortful control and parental substance use. Higher social fragmentation was associated with trajectories of moderately increasing cigarette use and both early and heavy alcohol use, while disorder was related to early but low-frequency cannabis use. Low socioeconomic deprivation was associated with trajectories of moderate and heavy increases in alcohol use. These associations mostly did not depend on parental substance use and adolescents’ effortful control. Taken together, the results indicate that neighborhood social disorganization in early adolescence relates to substance use up until young adulthood, but in diverse ways across different indicators of social disorganization and across substances. This highlights the potential value of targeted, place-based interventions to address youth substance use.

## Supplementary information

Supplementary Materials

## Footnotes

1 Adolescents’ educational levels may be a mediator of associations between effortful control and substance use outcomes. Because of this, the adjustment for adolescents’ educational levels in the models may have led to an overadjustment issue in the estimated associations. The negative associations between effortful control and odds of being in the heavy-increasing cigarette use trajectory (vs. stable low) and early-peaking alcohol use trajectory (vs. stable low) were significant before adjusting for adolescents’ educational levels (results available upon request).

## Declarations

### Data Sharing Declaration

The TRAILS survey data are available at https://www.trails.nl/, subject to the approval of the TRAILS committee and the data manager. The authors have no right to share the TRAILS data.
