# Supplementary material for: Neighborhood Social Disorganization in Early Adolescence and Substance Use Trajectories into Young Adulthood: The Moderating Role of Effortful Control and Parental Substance Use
Source: J Youth Adolesc. 2025 Oct 8;55(2):399–415. doi: 10.1007/s10964-025-02270-0 (PMC12894206; doi:10.1007/s10964-025-02270-0)
Supplement: Supplementary file 1 — Supplementary Materials [file 10964_2025_2270_MOESM1_ESM.docx]

**Online Supplementary Material for**

**Neighborhood Social Disorganization in Early Adolescence and Substance Use Trajectories into Young Adulthood: The Moderating Role of Effortful Control and Parental Substance Use**

**Table S1**

*Pairwise Spearman Correlations among the Study Variables*

| Variable | 1 | 2 | 3 | 4 | 5 | 6 | 7 | 8 | 9 | 10 | 11 | 12 | 13 |
| --- | --- | --- | --- | --- | --- | --- | --- | --- | --- | --- | --- | --- | --- |
| 1. Sex | 1.00 |  |  |  |  |  |  |  |  |  |  |  |  |
| 2. Ethnicity | -0.01 | 1.00 |  |  |  |  |  |  |  |  |  |  |  |
| 3. Parental divorce | -0.01 | 0.08** | 1.00 |  |  |  |  |  |  |  |  |  |  |
| 4. Family socioeconomic status | -0.03 | -0.16** | -0.25** | 1.00 |  |  |  |  |  |  |  |  |  |
| 5. Adolescents’ educational levels | -0.07** | -0.12** | -0.14** | 0.52** | 1.00 |  |  |  |  |  |  |  |  |
| 6. Parental internalizing problems | -0.02 | 0.00 | 0.24** | -0.10** | -0.07** | 1.00 |  |  |  |  |  |  |  |
| 7. Parental externalizing problems | -0.00 | 0.04 | 0.44** | -0.21** | -0.15** | 0.29** | 1.00 |  |  |  |  |  |  |
| 8. Social fragmentation | 0.01 | 0.18** | 0.16** | -0.07** | -0.04 | 0.03 | 0.12** | 1.00 |  |  |  |  |  |
| 9. Socioeconomic deprivation | 0.01 | 0.22** | 0.18** | -0.32** | -0.20** | 0.08** | 0.16** | 0.64** | 1.00 |  |  |  |  |
| 10. Disorder | -0.02 | 0.18** | 0.16** | -0.09** | -0.08** | 0.06** | 0.12** | 0.72** | 0.58** | 1.00 |  |  |  |
| 11. Parental cigarette use | 0.01 | -0.03 | 0.13** | -0.21** | -0.19** | 0.12** | 0.16** | 0.10** | 0.14** | 0.09** | 1.00 |  |  |
| 12. Parental alcohol use | 0.00 | -0.23** | -0.12** | 0.30** | 0.19** | -0.04 | -0.05* | 0.01 | -0.12** | -0.03 | 0.11** | 1.00 |  |
| 13. Parental addiction | 0.01 | 0.01 | 0.31** | -0.16** | -0.14** | 0.25** | 0.74** | 0.09** | 0.14** | 0.11** | 0.14** | -0.01 | 1.00 |
| 14. Adolescents’ effortful control | -0.18** | 0.02 | -0.13** | 0.16** | 0.41** | -0.12** | -0.10** | -0.03 | -0.03 | -0.01 | -0.14** | 0.01 | -0.08** |

Note: ** Correlation is significant at the 0.01 level (*P-value*); *Correlation is significant at the 0.05 level (*P-value*). The reference group for Sex was ‘female’, for Ethnicity was ‘Native’, for Parental divorce was ‘not divorced’, and for Parental addiction was ‘not addicted’. Adolescents’ educational level was treated as a continuous variable in the correlation test

**Table S2**

*Model-Fit Indices for Latent Class Growth Analysis Models on Cigarette Use*

| Model | AIC | BIC | aBIC | Entropy | BLRT P-value |
| --- | --- | --- | --- | --- | --- |
| Quadratic + linear |  |  |  |  |  |
| 1 class | 25594.52 | 25628.53 | 25609.47 | NA | NA |
| 2 classes | 20869.10 | 20925.78 | 20894.01 | 0.82 | 0 |
| 3 classes | 18939.40 | 19018.75 | 18974.27 | 0.82 | 0 |
| 4 classes | 18212.73 | 18314.75 | 18257.56 | 0.80 | 0 |
| Linear |  |  |  |  |  |
| 1 classes | 25715.53 | 25738.20 | 25725.49 | NA | NA |
| 2 classes | 20969.98 | 21009.65 | 20987.41 | 0.83 | 0 |
| 3 classes | 19048.58 | 19105.26 | 19073.49 | 0.82 | 0 |
| 4 classes | 18430.26 | 18503.94 | 18462.64 | 0.81 | 0 |

**Table S3**

*Model-Fit Indices for Latent Class Growth Analysis Models on Alcohol Use*

| Model | AIC | BIC | aBIC | Entropy | BLRT P-value |
| --- | --- | --- | --- | --- | --- |
| Quadratic + linear |  |  |  |  |  |
| 1 class | 71649.69 | 71666.66 | 71657.13 | NA | NA |
| 2 classes | 52423.83 | 52463.43 | 52441.19 | 0.94 | 0 |
| 3 classes | 47739.36 | 47801.59 | 47766.64 | 0.87 | 0 |
| 4 classes | 44823.45 | 44908.30 | 44860.64 | 0.87 | 0 |
| 5 classes | 43115.71 | 43223.18 | 43162.82 | 0.84 | 0 |
| Linear |  |  |  |  |  |
| 1 class | 75701.11 | 75712.42 | 75706.07 | NA | NA |
| 2 classes | 56001.89 | 56030.18 | 56014.29 | 0.94 | 0 |
| 3 classes | 51366.36 | 51411.61 | 51386.19 | 0.88 | 0 |
| 4 classes | 49574.04 | 49636.27 | 49601.32 | 0.89 | 0 |
| 5 classes | 47977.45 | 48056.65 | 48012.17 | 0.86 | 0 |

**Table S4**

*Model-Fit Indices for Latent Class Growth Analysis Models on Cannabis Use*

| Model | AIC | BIC | aBIC | Entropy | BLRT P-value |
| --- | --- | --- | --- | --- | --- |
| Quadratic + linear |  |  |  |  |  |
| 1 class | 18129.22 | 18163.19 | 18144.13 | NA | NA |
| 2 classes | 12248.96 | 12305.57 | 12273.80 | 0.41 | 0 |
| 3 classes | 11362.38 | 11441.64 | 11397.16 | 0.42 | 0 |
| 4 classes | 10680.70 | 10782.61 | 10725.42 | 0.79 | 0 |
| 5 classes | 10230.44 | 10354.99 | 10285.09 | 0.80 | 0 |
| 6 classes | 9907.01 | 10054.21 | 9971.61 | 0.80 | 0 |
| Linear |  |  |  |  |  |
| 1 classes | 18259.03 | 18293.00 | 18273.94 | NA | NA |
| 2 classes | 12337.93 | 12388.89 | 12360.29 | 0.41 | 0 |
| 3 classes | 11476.59 | 11544.53 | 11506.40 | 0.78 | 0 |
| 4 classes | 10852.11 | 10937.03 | 10889.37 | 0.79 | 0 |
| 5 classes | 10414.35 | 10516.26 | 10459.07 | 0.80 | 0 |
| 6 classes | 10172.38 | 10291.27 | 10224.55 | 0.78 | 0 |

**Table S5**

*Unstandardized Results from Multilevel Multinomial Regression Models for Membership in Cigarette Use Trajectories with Neighborhood-Level Indicators and Cross-Level Interaction Terms between Neighborhood-Level Indicators with Parental Cigarette Use and Adolescents’ Effortful Control (n = 2,229)*

| **Trajectory** | Model 1 | | | Model 2 | | | Model 3 | | |
| --- | --- | --- | --- | --- | --- | --- | --- | --- | --- |
| Variable | B (95% CI) | OR (95% CI) | *P-value* | B (95% CI) | OR (95% CI) | *P-value* | B (95% CI) | OR (95% CI) | *P-value* |
| **Moderate increasing (ref: stable low)** |  |  |  |  |  |  |  |  |  |
| Social fragmentation | **0.05 (0.01, 0.09)** | **1.05 (1.01, 1.10)** | **0.008** | **0.07 (0.01, 0.13)** | **1.07 (1.01, 1.14)** | **0.017** | **0.07 (0.01, 0.13)** | **1.07 (1.01, 1.14)** | **0.024** |
| Deprivation | 0.01 (-0.05, 0.07) | 1.01 (0.95, 1.07) | 0.778 | -0.05 (-0.13, 0.02) | 0.95 (0.88, 1.02) | 0.183 | -0.05 (-0.12, 0.02) | 0.95 (0.88, 1.02) | 0.193 |
| Disorder | 0.00 (-0.00, 0.01) | 1.00 (0.99, 1.01) | 0.155 | 0.00 (-0.01, 0.01) | 1.00 (0.99, 1.01) | 0.627 | 0.00 (-0.01, 0.01) | 1.00 (0.99, 1.01) | 0.642 |
| Parental cigarette use ^1^ | **0.02 (0.01, 0.04)** | **1.02 (1.01, 1.04)** | **0.007** | **0.02 (0.01, 0.04)** | **1.02 (1.01, 1.04)** | **0.007** | **0.02 (0.00, 0.04)** | **1.02 (1.00, 1.04)** | **0.010** |
| Effortful control ^1^ | **-0.22 (-0.42, -0.02)** | **0.80 (0.66, 0.98)** | **0.029** | **-0.21 (-0.42, -0.00)** | **0.81 (0.66, 0.99)** | **0.046** | **-0.21 (-0.42, -0.00)** | **0.81 (0.66, 0.99)** | **0.048** |
| Social fragmentation × Parental cigarette use |  |  |  |  |  |  | 0.00 (-0.00, 0.01) | 1.00 (0.99, 1.01) | 0.461 |
| Deprivation × Parental cigarette use |  |  |  |  |  |  | 0.00 (-0.01, 0.01) | 1.00 (0.99, 1.01) | 0.754 |
| Disorder × Parental cigarette use |  |  |  |  |  |  | 0.00 (-0.00, 0.00) | 1.00 (0.99, 1.00) | 0.545 |
| Social fragmentation × Effortful control |  |  |  |  |  |  | -0.01 (-0.12, 0.09) | 0.99 (0.89, 1.09) | 0.781 |
| Deprivation × Effortful control |  |  |  |  |  |  | -0.01 (-0.11, 0.09) | 0.99 (0.90, 1.09) | 0.848 |
| Disorder × Effortful control |  |  |  |  |  |  | 0.00 (-0.01, 0.02) | 1.00 (0.99, 1.02) | 0.634 |
| **Heavy increasing (ref: stable low)** |  |  |  |  |  |  |  |  |  |
| Social fragmentation | -0.01 (-0.06, 0.05) | 0.99 (0.94, 1.05) | 0.788 | 0.03 (-0.05, 0.10) | 1.03 (0.96, 1.11) | 0.462 | 0.04 (-0.03, 0.12) | 1.04 (0.97, 1.13) | 0.262 |
| Deprivation | -0.04 (-0.11, 0.02) | 0.96 (0.90, 1.02) | 0.197 | -0.07 (-0.15, 0.01) | 0.94 (0.86, 1.01) | 0.100 | -0.07 (-0.16, 0.02) | 0.93 (0.86, 1.02) | 0.121 |
| Disorder | -0.00 (-0.01, 0.01) | 1.00 (0.99, 1.01) | 0.792 | 0.00 (-0.01, 0.01) | 1.00 (0.99, 1.01) | 0.863 | 0.00 (-0.01, 0.01) | 1.00 (0.99, 1.01) | 0.972 |
| Parental cigarette use ^1^ | **0.06 (0.04, 0.07)** | **1.06 (1.04, 1.07)** | **0.000** | **0.06 (0.04, 0.07)** | **1.06 (1.04, 1.07)** | **0.000** | **0.06 (0.04, 0.07)** | **1.06 (1.04, 1.08)** | **0.000** |
| Effortful control ^1^ | -0.15 (-0.38, 0.07) | 0.86 (0.69, 1.07) | 0.179 | -0.14 (-0.34, 0.09) | 0.87 (0.70, 1.09) | 0.232 | -0.12 (-0.34, 0.10) | 0.89 (0.72, 1.11) | 0.303 |
| Social fragmentation × Parental cigarette use |  |  |  |  |  |  | 0.00 (-0.01, 0.01) | 1.00 (0.99, 1.01) | 0.616 |
| Deprivation × Parental cigarette use |  |  |  |  |  |  | -0.00 (-0.01, 0.00) | 1.00 (0.99, 1.00) | 0.423 |
| Disorder × Parental cigarette use |  |  |  |  |  |  | 0.00 (-0.00, 0.00) | 1.00 (0.99, 1.00) | 0.836 |
| Social fragmentation × Effortful control |  |  |  |  |  |  | 0.10 (-0.01, 0.22) | 1.11 (0.99, 1.25) | 0.083 |
| Deprivation × Effortful control |  |  |  |  |  |  | -0.08 (-0.19, 0.02) | 0.92 (0.83, 1.02) | 0.110 |
| Disorder × Effortful control |  |  |  |  |  |  | -0.00 (-0.01, 0.01) | 1.00 (0.99, 1.01) | 0.713 |

Note: B = unstandardized regression coefficient; OR = odds ratio; 95% CI = 95% confidence interval; ref = the reference group. ^1^ Both parental cigarette use and adolescents’ effortful control were controlled in Model 1 for each neighborhood-level indicator, and the coefficients of these two variables from Model 1 for social fragmentation were reported in the corresponding column for Model 1 in this table (similar results on these coefficients were found in Model 1 for socioeconomic deprivation and disorder). The estimates with the *p-values<0.05* are shown in bold

**Table S6**

*Unstandardized Results from Multilevel Multinomial Regression Models for Membership in Alcohol Use Trajectories with Neighborhood-Level Indicators and Cross-Level Interaction Terms between Neighborhood-Level Indicators with Parental Alcohol Use and Adolescents’ Effortful Control (n = 2,229)*

| **Trajectory** | Model 1 | | | Model 2 | | | Model 3 | | |
| --- | --- | --- | --- | --- | --- | --- | --- | --- | --- |
| Variable | B (95% CI) | OR (95% CI) | *P-value* | B (95% CI) | OR (95% CI) | *P-value* | B (95% CI) | OR (95% CI) | *P-value* |
| **Moderate increasing (ref: stable low)** |  |  |  |  |  |  |  |  |  |
| Social fragmentation | 0.00 (-0.05, 0.06) | 1.00 (0.95, 1.06) | 0.886 | 0.06 (-0.02, 0.13) | 1.06 (0.98, 1.14) | 0.119 | 0.06 (-0.02, 0.14) | 1.06 (0.98, 1.15) | 0.140 |
| Deprivation | **-0.06 (-0.12, -0.00)** | **0.94 (0.88, 0.99)** | **0.036** | **-0.12 (-0.18, -0.05)** | **0.89 (0.83, 0.95)** | **0.001** | **-0.11 (-0.19, -0.04)** | **0.89 (0.83, 0.96)** | **0.003** |
| Disorder | 0.00 (-0.01, 0.01) | 1.00 (0.99, 1.01) | 0.953 | 0.00 (-0.00, 0.01) | 1.00 (1.00, 1.01) | 0.378 | 0.00 (-0.00, 0.01) | 1.00 (0.99, 1.01) | 0.638 |
| Parental alcohol use ^1^ | **0.07 (0.04, 0.10)** | **1.07 (1.04, 1.10)** | **0.000** | **0.07 (0.03, 0.10)** | **1.07 (1.04, 1.10)** | **0.000** | **0.07 (0.04, 0.10)** | **1.07 (1.04, 1.10)** | **0.000** |
| Effortful control ^1^ | -0.15 (-0.36, 0.06) | 0.86 (0.70, 1.07) | 0.173 | -0.12 (-0.34, 0.09) | 0.88 (0.71, 1.09) | 0.256 | -0.13 (-0.34, 0.07) | 0.88 (0.71, 1.08) | 0.208 |
| Social fragmentation × Parental alcohol use |  |  |  |  |  |  | 0.01 (-0.00, 0.03) | 1.01 (1.00, 1.03) | 0.125 |
| Deprivation × Parental alcohol use |  |  |  |  |  |  | 0.00 (-0.01, 0.01) | 1.00 (0.99, 1.01) | 0.994 |
| Disorder × Parental alcohol use |  |  |  |  |  |  | -0.00 (-0.00, 0.00) | 1.00 (0.99, 1.00) | 0.086 |
| Social fragmentation × Effortful control |  |  |  |  |  |  | -0.05 (-0.14, 0.04) | 0.95 (0.86, 1.04) | 0.249 |
| Deprivation × Effortful control |  |  |  |  |  |  | -0.08 (-0.18, 0.02) | 0.93 (0.84, 1.02) | 0.124 |
| Disorder × Effortful control |  |  |  |  |  |  | **0.02 (0.01, 0.03)** | **1.02 (1.01, 1.03)** | **0.001** |
| **Heavy increasing (ref: stable low)** |  |  |  |  |  |  |  |  |  |
| Social fragmentation | 0.04 (-0.04, 0.12) | 1.04 (0.96, 1.12) | 0.324 | **0.15 (0.06, 0.25)** | **1.16 (1.06, 1.28)** | **0.002** | **0.15 (0.05, 0.25)** | **1.16 (1.05, 1.28)** | **0.003** |
| Deprivation | -0.07 (-0.17, 0.03) | 0.93 (0.84, 1.03) | 0.185 | **-0.14 (-0.26, -0.03)** | **0.89 (0.78, 0.97)** | **0.014** | **-0.14 (-0.25, -0.02)** | **0.87 (0.78, 0.99)** | **0.019** |
| Disorder | -0.00 (-0.01, 0.01) | 1.00 (1.00, 1.01) | 0.629 | -0.01 (-0.02, 0.01) | 0.99 (0.98, 1.01) | 0.322 | -0.01 (-0.02, 0.00) | 0.99 (0.99, 1.00) | 0.241 |
| Parental alcohol use ^1^ | **0.08 (0.05, 0.12)** | **1.09 (1.05, 1.12)** | **0.000** | **0.08 (0.05, 0.12)** | **1.09 (1.05, 1.12)** | **0.000** | **0.08 (0.05, 0.12)** | **1.09 (1.05, 1.12)** | **0.000** |
| Effortful control ^1^ | -0.06 (-0.36, 0.24) | 0.94 (0.69, 1.26) | 0.684 | -0.02 (-0.32, 0.28) | 0.98 (0.72, 1.33) | 0.900 | -0.08 (-0.40, 0.24) | 0.92 (0.67, 1.27) | 0.628 |
| Social fragmentation × Parental alcohol use |  |  |  |  |  |  | 0.01 (-0.01, 0.04) | 1.01 (0.99, 1.04) | 0.206 |
| Deprivation × Parental alcohol use |  |  |  |  |  |  | -0.01 (-0.03, 0.01) | 0.99 (0.97, 1.01) | 0.483 |
| Disorder × Parental alcohol use |  |  |  |  |  |  | -0.00 (-0.00, 0.00) | 1.00 (1.00, 1.00) | 0.233 |
| Social fragmentation × Effortful control |  |  |  |  |  |  | 0.02 (-0.12, 0.16) | 1.02 (0.89, 1.18) | 0.755 |
| Deprivation × Effortful control |  |  |  |  |  |  | -0.07 (-0.22, 0.07) | 0.93 (0.81, 1.08) | 0.332 |
| Disorder × Effortful control |  |  |  |  |  |  | -0.00 (-0.02, 0.02) | 1.00 (0.98, 1.02) | 0.698 |
| **Early peaking (ref: stable low)** |  |  |  |  |  |  |  |  |  |
| Social fragmentation | 0.07 (-0.00, 0.14) | 1.07 (1.00, 1.15) | 0.053 | **0.10 (0.00, 0.20)** | **1.11 (1.00, 1.22)** | **0.047** | 0.09 (-0.02, 0.20) | 1.09 (0.98, 1.22) | 0.128 |
| Deprivation | 0.02 (-0.06, 0.09) | 1.02 (0.94, 1.10) | 0.654 | -0.06 (-0.16, 0.04) | 0.94 (0.85, 1.04) | 0.231 | -0.04 (-0.15, 0.06) | 0.96 (0.86, 1.06) | 0.394 |
| Disorder | 0.00 (-0.00, 0.02) | 1.00 (0.99, 1.02) | 0.266 | 0.00 (-0.01, 0.01) | 1.00 (0.99, 1.01) | 0.792 | 0.00 (-0.01, 0.01) | 1.00 (0.99, 1.01) | 0.856 |
| Parental alcohol use ^1^ | **0.04 (0.01, 0.08)** | **1.05 (1.01, 1.08)** | **0.006** | **0.04 (0.01, 0.08)** | **1.05 (1.01, 1.08)** | **0.007** | **0.04 (0.01, 0.08)** | **1.04 (1.01, 1.08)** | **0.009** |
| Effortful control ^1^ | -0.19 (-0.49, 0.11) | 0.83 (0.61, 1.12) | 0.223 | -0.17 (-0.47, 0.13) | 0.84 (0.62, 1.14) | 0.258 | -0.18 (-0.46, 0.11) | 0.84 (0.63, 1.12) | 0.228 |
| Social fragmentation × Parental alcohol use |  |  |  |  |  |  | -0.00 (-0.02, 0.01) | 1.00 (0.99, 1.01) | 0.528 |
| Deprivation × Parental alcohol use |  |  |  |  |  |  | 0.00 (-0.01, 0.02) | 1.00 (0.99, 1.02) | 0.619 |
| Disorder × Parental alcohol use |  |  |  |  |  |  | 0.00 (-0.00, 0.00) | 1.00 (1.00, 1.00) | 0.712 |
| Social fragmentation × Effortful control |  |  |  |  |  |  | -0.12 (-0.26, 0.02) | 0.89 (0.77, 1.02) | 0.096 |
| Deprivation × Effortful control |  |  |  |  |  |  | 0.04 (-0.08, 0.16) | 1.04 (0.92, 1.18) | 0.542 |
| Disorder × Effortful control |  |  |  |  |  |  | 0.01 (-0.02, 0.03) | 1.01 (0.98, 1.03) | 0.551 |

Note: B = unstandardized regression coefficient; OR = odds ratio; 95% CI = 95% confidence interval; ref = the reference group. ^1^ Both parental alcohol use and adolescents’ effortful control were controlled in Model 1 for each neighborhood-level indicator, and the coefficients of these two variables from Model 1 for social fragmentation were reported in the corresponding column for Model 1 in this table (similar results on these coefficients were found in Model 1 for socioeconomic deprivation and disorder). The estimates with the *p-values<0.05* are shown in bold

**Table S7**

*Unstandardized Results from Multilevel Multinomial Regression Models for Membership in Cannabis Use Trajectories with Neighborhood-Level Indicators and Cross-Level Interaction Terms between Neighborhood-Level Indicators with Parental Addiction and Adolescents’ Effortful Control (n = 2,229)*

| **Trajectory** | Model 1 | | | Model 2 | | | Model 3 | | |
| --- | --- | --- | --- | --- | --- | --- | --- | --- | --- |
| Variable | B (95% CI) | OR (95% CI) | *P-value* | B (95% CI) | OR (95% CI) | *P-value* | B (95% CI) | OR (95% CI) | *P-value* |
| **Low (ref: never use)** |  |  |  |  |  |  |  |  |  |
| Social fragmentation | 0.07 (-0.01, 0.14) | 1.07 (0.99, 1.15) | 0.074 |  |  |  | 0.05 (-0.03, 0.13) | 1.05 (0.97, 1.14) | 0.257 |
| Deprivation | -0.00 (-0.10, 0.09) | 1.00 (0.91, 1.09) | 0.930 |  |  |  | -0.02 (-0.11, 0.08) | 0.98 (0.89, 1.08) | 0.712 |
| Disorder | **0.01 (0.00, 0.02)** | **1.01 (1.00, 1.02)** | **0.023** |  |  |  | 0.01 (0.00, 0.02) | 1.01 (1.00, 1.02) | 0.061 |
| Parental addiction ^1^ | 0.34 (-0.86, 1.53) | 1.40 (0.42, 4.63) | 0.581 |  |  |  | 0.14 (-1.12, 1.39) | 1.15 (0.33, 4.02) | 0.831 |
| Effortful control ^1^ | -0.26 (-0.57, 0.06) | 0.77 (0.57, 1.06) | 0.108 |  |  |  | -0.26 (-0.59, 0.07) | 0.77 (0.55, 1.07) | 0.118 |
| Social fragmentation × Parental addiction |  |  |  |  |  |  | **0.21 (0.01, 0.41)** | **1.24 (1.01, 1.51)** | **0.039** |
| Deprivation × Parental addiction |  |  |  |  |  |  | 0.19 (-0.10, 0.47) | 1.20 (0.90, 1.60) | 0.210 |
| Disorder × Parental addiction |  |  |  |  |  |  | 0.02 (-0.02, 0.06) | 1.02 (0.98, 1.07) | 0.355 |
| Social fragmentation × Effortful control |  |  |  |  |  |  | -0.04 (-0.15, 0.06) | 0.96 (0.86, 1.06) | 0.413 |
| Deprivation × Effortful control |  |  |  |  |  |  | -0.07 (-0.20, 0.06) | 0.93 (0.82, 1.06) | 0.278 |
| Disorder × Effortful control |  |  |  |  |  |  | -0.00 (-0.02, 0.01) | 1.00 (0.98, 1.01) | 0.574 |
| **Early increase (ref: never use)** |  |  |  |  |  |  |  |  |  |
| Social fragmentation | 0.02 (-0.08, 0.11) | 1.02 (0.92, 1.12) | 0.753 |  |  |  | 0.00 (-0.12, 0.13) | 1.00 (0.88, 1.14) | 0.977 |
| Deprivation | -0.02 (-0.14, 0.11) | 0.98 (0.87, 1.11) | 0.788 |  |  |  | -0.03 (-0.16, 0.11) | 0.97 (0.85, 1.11) | 0.687 |
| Disorder | 0.01 (-0.01, 0.02) | 1.01 (0.99, 1.02) | 0.437 |  |  |  | 0.00 (-0.01, 0.02) | 1.00 (0.99, 1.02) | 0.609 |
| Parental addiction ^1^ | -0.67 (-2.24, 0.91) | 0.51 (0.11, 2.47) | 0.406 |  |  |  | -0.66 (-2.24, 0.93) | 0.52 (0.11, 2.52) | 0.416 |
| Effortful control ^1^ | -0.20 (-0.70, 0.30) | 0.82 (0.50, 1.34) | 0.428 |  |  |  | -0.20 (-0.70, 0.30) | 0.82 (0.50, 1.34) | 0.424 |
| Social fragmentation × Parental addiction |  |  |  |  |  |  | -0.08 (-0.33, 0.16) | 0.92 (0.72, 1.17) | 0.502 |
| Deprivation × Parental addiction |  |  |  |  |  |  | -0.10 (-0.48, 0.29) | 0.91 (0.62, 1.33) | 0.617 |
| Disorder × Parental addiction |  |  |  |  |  |  | 0.00 (-0.03, 0.04) | 1.00 (0.97, 1.04) | 0.900 |
| Social fragmentation × Effortful control |  |  |  |  |  |  | -0.09 (-0.27, 0.09) | 0.91 (0.76, 1.09) | 0.313 |
| Deprivation × Effortful control |  |  |  |  |  |  | -0.17 (-0.35, 0.01) | 0.85 (0.71, 1.01) | 0.069 |
| Disorder × Effortful control |  |  |  |  |  |  | -0.02 (-0.04, 0.01) | 0.98 (0.96, 1.01) | 0.179 |
| **Peaking (ref: never use)** |  |  |  |  |  |  |  |  |  |
| Social fragmentation | 0.07 (-0.02, 0.15) | 1.07 (0.98, 1.16) | 0.129 |  |  |  | 0.06 (-0.04, 0.16) | 1.06 (0.96, 1.17) | 0.243 |
| Deprivation | 0.07 (-0.03, 0.18) | 1.07 (0.97, 1.19) | 0.181 |  |  |  | 0.07 (-0.04, 0.18) | 1.07 (0.96, 1.20) | 0.217 |
| Disorder | 0.01 (-0.00, 0.02) | 1.01 (0.99, 1.02) | 0.087 |  |  |  | 0.01 (-0.00, 0.02) | 1.01 (0.99, 1.02) | 0.118 |
| Parental addiction ^1^ | -0.45 (-2.12, 1.22) | 0.64 (0.12, 3.40) | 0.598 |  |  |  | -0.96 (-3.03, 1.12) | 0.38 (0.05, 3.05) | 0.365 |
| Effortful control ^1^ | **-0.50 (-0.97, -0.02)** | **0.61 (0.38, 0.98)** | **0.039** |  |  |  | **-0.56 (-1.04, -0.08)** | **0.57 (0.35, 0.93)** | **0.023** |
| Social fragmentation × Parental addiction |  |  |  |  |  |  | 0.30 (-0.05, 0.65) | 1.35 (0.95, 1.92) | 0.096 |
| Deprivation × Parental addiction |  |  |  |  |  |  | 0.19 (-0.14, 0.52) | 1.21 (0.87, 1.68) | 0.267 |
| Disorder × Parental addiction |  |  |  |  |  |  | 0.05 (-0.02, 0.13) | 1.05 (0.98, 1.14) | 0.187 |
| Social fragmentation × Effortful control |  |  |  |  |  |  | 0.06 (-0.06, 0.18) | 1.06 (0.94, 1.20) | 0.333 |
| Deprivation × Effortful control |  |  |  |  |  |  | 0.03 (-0.11, 0.18) | 1.03 (0.89, 1.19) | 0.668 |
| Disorder × Effortful control |  |  |  |  |  |  | 0.00 (-0.01, 0.02) | 1.00 (0.99, 1.02) | 0.612 |
| **Late increase (ref: never use)** |  |  |  |  |  |  |  |  |  |
| Social fragmentation | 0.09 (-0.06, 0.24) | 1.09 (0.94, 1.27) | 0.243 |  |  |  | 0.07 (-0.11, 0.26) | 1.08 (0.90, 1.29) | 0.433 |
| Deprivation | 0.05 (-0.14, 0.24) | 1.05 (0.87, 1.27) | 0.608 |  |  |  | 0.01 (-0.16, 0.18) | 1.01 (0.85, 1.20) | 0.888 |
| Disorder | 0.01 (-0.01, 0.02) | 1.01 (0.99, 1.02) | 0.262 |  |  |  | 0.01 (-0.00, 0.02) | 1.01 (0.99, 1.02) | 0.169 |
| Parental addiction ^1^ | -0.78 (-2.63, 1.07) | 0.46 (0.07, 2.93) | 0.411 |  |  |  | -0.84 (-2.75, 1.07) | 0.43 (0.06, 2.92) | 0.389 |
| Effortful control ^1^ | -0.25 (-0.94, 0.44) | 0.78 (0.39, 1.55) | 0.474 |  |  |  | -0.19 (-0.91, 0.52) | 0.82 (0.40, 1.69) | 0.596 |
| Social fragmentation × Parental addiction |  |  |  |  |  |  | 0.02 (-0.46, 0.51) | 1.02 (0.63, 1.66) | 0.924 |
| Deprivation × Parental addiction |  |  |  |  |  |  | 0.22 (-0.76, 1.19) | 1.24 (0.47, 3.29) | 0.661 |
| Disorder × Parental addiction |  |  |  |  |  |  | -0.02 (-0.08, 0.03) | 0.98 (0.92, 1.03) | 0.373 |
| Social fragmentation × Effortful control |  |  |  |  |  |  | -0.08 (-0.30, 0.14) | 0.92 (0.74, 1.16) | 0.480 |
| Deprivation × Effortful control |  |  |  |  |  |  | -0.15 (-0.37, 0.09) | 0.87 (0.69, 1.09) | 0.228 |
| Disorder × Effortful control |  |  |  |  |  |  | -0.01 (-0.03, 0.02) | 0.99 (0.97, 1.02) | 0.536 |

Note: B = unstandardized regression coefficient; OR = odds ratio; 95% CI = 95% confidence interval; ref = the reference group. ^1^ Both parental addiction and adolescents’ effortful control were controlled in Model 1 for each neighborhood-level indicator, and the coefficients of these two variables from Model 1 for social fragmentation were reported in the corresponding column for Model 1 in this table (similar results on these coefficients were found in Model 1 for socioeconomic deprivation and disorder). For Model 3 (adding interactions into Model 1, instead of Model 2), results for parental addiction and adolescents’ effortful control were from the model for social fragmentation, which had a significant interaction term with parental addiction. The estimates with the *p-values<0.05* are shown in bold

**Table S8**

*Unstandardized Results of the First Sensitivity Analysis Refitting Model 3 (for Cigarette Use Trajectories) by Adding the Cross-Level Interaction Terms into Model 1 (n = 2,229)*

|  | B (95% CI) | OR (95% CI) | *P-value* |
| --- | --- | --- | --- |
| **Moderate increasing (ref: stable low)** |  |  |  |
| Social fragmentation | **0.05 (0.01, 0.09)** | **1.05 (1.01, 1.10)** | **0.010** |
| Deprivation | 0.01 (-0.05, 0.07) | 1.01 (0.95, 1.07) | 0.786 |
| Disorder | 0.00 (-0.00, 0.01) | 1.00 (0.99, 1.01) | 0.149 |
| Parental cigarette use ^1^ | **0.02 (0.00, 0.04)** | **1.02 (1.00, 1.04)** | **0.011** |
| Effortful control ^1^ | **-0.22 (-0.42, -0.02)** | **0.80 (0.66, 0.98)** | **0.035** |
| Social fragmentation × Parental cigarette use | 0.00 (-0.00, 0.01) | 1.00 (0.99, 1.01) | 0.350 |
| Deprivation × Parental cigarette use | 0.00 (-0.01, 0.01) | 1.00 (0.99, 1.01) | 0.578 |
| Disorder × Parental cigarette use | 0.00 (-0.00, 0.00) | 1.00 (1.00, 1.00) | 0.780 |
| Social fragmentation × Effortful control | -0.01 (-0.08, 0.07) | 0.99 (0.92, 1.07) | 0.852 |
| Deprivation × Effortful control | -0.00 (-0.08, 0.07) | 1.00 (0.92, 1.08) | 0.905 |
| Disorder × Effortful control | 0.00 (-0.01, 0.01) | 1.00 (0.99, 1.01) | 0.886 |
| **Heavy increasing (ref: stable low)** |  |  |  |
| Social fragmentation | 0.00 (-0.05, 0.06) | 1.00 (0.95, 1.06) | 0.930 |
| Deprivation | -0.04 (-0.11, 0.03) | 0.96 (0.90, 1.03) | 0.255 |
| Disorder | -0.00 (-0.01, 0.01) | 1.00 (0.99, 1.01) | 0.880 |
| Parental cigarette use ^1^ | **0.06 (0.04, 0.07)** | **1.06 (1.04, 1.08)** | **0.000** |
| Effortful control ^1^ | -0.15 (-0.37, 0.07) | 0.86 (0.69, 1.07) | 0.174 |
| Social fragmentation × Parental cigarette use | -0.00 (-0.01, 0.00) | 1.00 (0.99, 1.01) | 0.763 |
| Deprivation × Parental cigarette use | -0.00 (-0.01, 0.00) | 1.00 (0.99, 1.00) | 0.462 |
| Disorder × Parental cigarette use | 0.00 (-0.00, 0.00) | 1.00 (1.00, 1.00) | 0.645 |
| Social fragmentation × Effortful control | 0.03 (-0.05, 0.11) | 1.03 (0.96, 1.12) | 0.419 |
| Deprivation × Effortful control | -0.02 (-0.10, 0.05) | 0.98 (0.91, 1.05) | 0.553 |
| Disorder × Effortful control | 0.00 (-0.01, 0.01) | 1.00 (0.99, 1.01) | 0.967 |

Note: B = unstandardized regression coefficient; OR = odds ratio; 95% CI = 95% confidence interval; ref = the reference group. ^1^ Both parental cigarette use and adolescents’ effortful control were controlled in the model for each neighborhood-level indicator, and the coefficients of these two variables from the model for social fragmentation were reported here (similar results on these coefficients were found in models for socioeconomic deprivation and disorder). The estimates with the *p-values<0.05* are shown in bold

**Table S9**

*Unstandardized* *Results of the First Sensitivity Analysis Refitting Model 3 (for Alcohol Use Trajectories) by Adding the Cross-Level Interaction Terms into Model 1 (n = 2,229)*

|  | B (95% CI) | OR (95% CI) | *P-value* |
| --- | --- | --- | --- |
| **Moderate increasing (ref: stable low)** |  |  |  |
| Social fragmentation | 0.00 (-0.06, 0.06) | 1.00 (0.94, 1.06) | 0.953 |
| Deprivation | **-0.06 (-0.12, -0.00)** | **0.94 (0.88, 0.99)** | **0.039** |
| Disorder | 0.00 (-0.01, 0.01) | 1.00 (0.99, 1.01) | 0.946 |
| Parental alcohol use ^1^ | **0.07 (0.04, 0.10)** | **1.07 (1.04, 1.10)** | **0.000** |
| Effortful control ^1^ | -0.14 (-0.36, 0.07) | 0.86 (0.70, 1.07) | 0.180 |
| Social fragmentation × Parental alcohol use | 0.01 (-0.00, 0.02) | 1.01 (1.00, 1.02) | 0.203 |
| Deprivation × Parental alcohol use | 0.00 (-0.01, 0.01) | 1.00 (0.99, 1.02) | 0.685 |
| Disorder × Parental alcohol use | 0.00 (-0.00, 0.00) | 1.00 (1.00, 1.00) | 0.866 |
| Social fragmentation × Effortful control | -0.00 (-0.07, 0.07) | 1.00 (0.93, 1.07) | 0.948 |
| Deprivation × Effortful control | -0.02 (-0.11, 0.07) | 0.98 (0.90, 1.07) | 0.625 |
| Disorder × Effortful control | **0.01 (0.00, 0.02)** | **1.01 (1.00, 1.02)** | **0.027** |
| **Heavy increasing (ref: stable low)** |  |  |  |
| Social fragmentation | 0.03 (-0.05, 0.11) | 1.04 (0.96, 1.12) | 0.408 |
| Deprivation | -0.07 (-0.17, 0.03) | 0.94 (0.84, 1.03) | 0.190 |
| Disorder | -0.00 (-0.01, 0.01) | 1.00 (0.99, 1.01) | 0.533 |
| Parental alcohol use ^1^ | **0.09 (0.05, 0.12)** | **1.09 (1.05, 1.13)** | **0.000** |
| Effortful control ^1^ | -0.06 (-0.35, 0.24) | 0.94 (0.70, 1.27) | 0.703 |
| Social fragmentation × Parental alcohol use | 0.01 (-0.01, 0.02) | 1.01 (0.99, 1.02) | 0.311 |
| Deprivation × Parental alcohol use | -0.00 (-0.02, 0.01) | 1.00 (0.98, 1.02) | 0.648 |
| Disorder × Parental alcohol use | 0.00 (-0.00, 0.00) | 1.00 (1.00, 1.00) | 0.733 |
| Social fragmentation × Effortful control | -0.04 (-0.14, 0.07) | 0.97 (0.87, 1.07) | 0.497 |
| Deprivation × Effortful control | -0.06 (-0.17, 0.04) | 0.94 (0.84, 1.04) | 0.240 |
| Disorder × Effortful control | -0.01 (-0.02, 0.01) | 0.99 (0.98, 1.01) | 0.375 |
| **Early peaking (ref: stable low)** |  |  |  |
| Social fragmentation | 0.07 (-0.01, 0.14) | 1.07 (0.99, 1.15) | 0.084 |
| Deprivation | 0.02 (-0.06, 0.10) | 1.02 (0.94, 1.11) | 0.636 |
| Disorder | 0.00 (-0.00, 0.02) | 1.00 (1.00, 1.02) | 0.346 |
| Parental alcohol use ^1^ | **0.05 (0.02, 0.08)** | **1.05 (1.02, 1.08)** | **0.004** |
| Effortful control ^1^ | -0.17 (-0.47, 0.13) | 0.84 (0.63, 1.14) | 0.272 |
| Social fragmentation × Parental alcohol use | -0.00 (-0.01, 0.01) | 1.00 (0.99, 1.01) | 0.861 |
| Deprivation × Parental alcohol use | 0.00 (-0.01, 0.01) | 1.00 (0.99, 1.01) | 0.891 |
| Disorder × Parental alcohol use | 0.00 (-0.00, 0.00) | 1.00 (1.00, 1.00) | 0.782 |
| Social fragmentation × Effortful control | -0.06 (-0.15, 0.03) | 0.94 (0.86, 1.03) | 0.184 |
| Deprivation × Effortful control | -0.01 (-0.12, 0.10) | 0.99 (0.88, 1.10) | 0.834 |
| Disorder × Effortful control | -0.00 (-0.02, 0.01) | 1.00 (0.98, 1.01) | 0.898 |

Note: B = unstandardized regression coefficient; OR = odds ratio; 95% CI = 95% confidence interval; ref = the reference group. ^1^ Both parental alcohol use and adolescents’ effortful control were controlled in the model for each neighborhood-level indicator, and the coefficients of these two variables from the model for social fragmentation were reported here (similar results on these coefficients were found in models for socioeconomic deprivation and disorder). The estimates with the *p-values<0.05* are shown in bold

**Table S10**

*Unstandardized Results of the Second Sensitivity Analysis Refitting Models 1-2 (for Cigarette Use Trajectories) without Adjusting for Effortful Control and Parental Cigarette Use (n = 2,229)*

| **Trajectory** | Model 1 | | | Model 2 | | |
| --- | --- | --- | --- | --- | --- | --- |
| Variable | B (95% CI) | OR (95% CI) | *P-value* | B (95% CI) | OR (95% CI) | *P-value* |
| **Moderate increasing (ref: stable low)** |  |  |  |  |  |  |
| Social fragmentation | **0.06 (0.02, 0.10)** | **1.06 (1.02, 1.10)** | **0.003** | **0.08 (0.02, 0.14)** | **1.08 (1.02, 1.15)** | **0.009** |
| Deprivation | 0.01 (-0.05, 0.07) | 1.01 (0.95, 1.07) | 0.739 | -0.06 (-0.13, 0.02) | 0.94 (0.88, 1.02) | 0.126 |
| Disorder | 0.00 (-0.00, 0.01) | 1.00 (1.00, 1.01) | 0.101 | 0.00 (-0.01, 0.01) | 1.00 (0.99, 1.01) | 0.560 |
| **Heavy increasing (ref: stable low)** |  |  |  |  |  |  |
| Social fragmentation | 0.01 (-0.05, 0.06) | 1.01 (0.95, 1.07) | 0.751 | 0.04 (-0.04, 0.11) | 1.04 (0.96, 1.12) | 0.351 |
| Deprivation | -0.03 (-0.09, 0.04) | 0.97 (0.91, 1.04) | 0.378 | -0.06 (-0.14, 0.02) | 0.94 (0.86, 1.02) | 0.113 |
| Disorder | 0.00 (-0.01, 0.01) | 1.00 (0.99, 1.01) | 0.766 | 0.00 (-0.01, 0.01) | 1.00 (0.99, 1.01) | 0.647 |

Note: B = unstandardized regression coefficient; OR = odds ratio; 95% CI = 95% confidence interval; ref = the reference group. The estimates with the *p-values<0.05* are shown in bold

**Table S11**

*Unstandardized Results of the Second Sensitivity Analysis Refitting Models 1-2 (for Alcohol Use Trajectories) without Adjusting for Effortful Control and Parental Alcohol Use (n = 2,229)*

| **Trajectory** | Model 1 | | | Model 2 | | |
| --- | --- | --- | --- | --- | --- | --- |
| Variable | B (95% CI) | OR (95% CI) | *P-value* | B (95% CI) | OR (95% CI) | *P-value* |
| **Moderate increasing (ref: stable low)** |  |  |  |  |  |  |
| Social fragmentation | 0.02 (-0.04, 0.07) | 1.02 (0.96, 1.08) | 0.538 | **0.08 (0.01, 0.14)** | **1.08 (1.01, 1.16)** | **0.032** |
| Deprivation | **-0.06 (-0.12, -0.00)** | **0.94 (0.89, 0.99)** | **0.047** | **-0.12 (-0.19, -0.06)** | **0.88 (0.82, 0.94)** | **0.000** |
| Disorder | 0.00 (-0.00, 0.01) | 1.00 (1.00, 1.01) | 0.733 | 0.00 (-0.00, 0.01) | 1.00 (0.99, 1.01) | 0.398 |
| **Heavy increasing (ref: stable low)** |  |  |  |  |  |  |
| Social fragmentation | 0.05 (-0.02, 0.13) | 1.05 (0.98, 1.14) | 0.171 | **0.17 (0.07, 0.26)** | **1.18 (1.08, 1.30)** | **0.000** |
| Deprivation | -0.06 (-0.16, 0.03) | 0.94 (0.85, 1.04) | 0.205 | **-0.15 (-0.26, -0.04)** | **0.86 (0.77, 0.96)** | **0.006** |
| Disorder | -0.00 (-0.01, 0.01) | 1.00 (0.99, 1.01) | 0.806 | -0.01 (-0.02, 0.01) | 0.99 (0.98, 1.01) | 0.343 |
| **Early peaking (ref: stable low)** |  |  |  |  |  |  |
| Social fragmentation | **0.08 (0.00, 0.15)** | **1.08 (1.00, 1.16)** | **0.035** | **0.11 (0.01, 0.21)** | **1.12 (1.01, 1.24)** | **0.025** |
| Deprivation | 0.02 (-0.06, 0.09) | 1.02 (0.94, 1.10) | 0.679 | -0.07 (-0.17, 0.03) | 0.93 (0.84, 1.03) | 0.176 |
| Disorder | 0.01 (-0.00, 0.02) | 1.01 (1.00, 1.02) | 0.235 | 0.00 (-0.01, 0.01) | 1.00 (0.99, 1.01) | 0.810 |

Note: B = unstandardized regression coefficient; OR = odds ratio; 95% CI = 95% confidence interval; ref = the reference group. The estimates with the *p-values<0.05* are shown in bold. The positive association between neighborhood social fragmentation and membership in the moderate-increasing alcohol use trajectory (vs. stable low) turned significant after removing effortful control and parental alcohol use (mainly parental alcohol use) from Model 2. As neighborhood-level indicators and parental alcohol use were both measured at *T_1_*, there is no way to determine whether parental alcohol use functioned as a mediator or a confounder in explaining this association

**Table S12**

*Unstandardized Results of the Second Sensitivity Analysis Refitting Model 1 (For Cannabis Use Trajectories) without Adjusting for Effortful Control and Parental Addiction (n = 2,229)*

|  | B (95% CI) | OR (95% CI) | *P-value* |
| --- | --- | --- | --- |
| **Low (ref: never use)** |  |  |  |
| Social fragmentation | 0.07 (-0.01, 0.14) | 1.07 (0.99, 1.15) | 0.077 |
| Deprivation | -0.01 (-0.10, 0.08) | 0.99 (0.91, 1.08) | 0.800 |
| Disorder | **0.01 (0.00, 0.02)** | **1.01 (1.00, 1.02)** | **0.025** |
| **Early increase (ref: never use)** |  |  |  |
| Social fragmentation | 0.02 (-0.08, 0.11) | 1.02 (0.92, 1.12) | 0.726 |
| Deprivation | -0.02 (-0.14, 0.10) | 0.98 (0.87, 1.10) | 0.727 |
| Disorder | 0.01 (-0.01, 0.02) | 1.01 (0.99, 1.02) | 0.441 |
| **Peaking (ref: never use)** |  |  |  |
| Social fragmentation | 0.07 (-0.01, 0.16) | 1.08 (0.99, 1.17) | 0.096 |
| Deprivation | 0.06 (-0.04, 0.17) | 1.07 (0.96, 1.18) | 0.215 |
| Disorder | 0.01 (-0.00, 0.02) | 1.01 (1.00, 1.02) | 0.091 |
| **Late increase (ref: never use)** |  |  |  |
| Social fragmentation | 0.10 (-0.05, 0.24) | 1.10 (0.96, 1.27) | 0.186 |
| Deprivation | 0.05 (-0.11, 0.22) | 1.06 (0.89, 1.25) | 0.522 |
| Disorder | 0.01 (-0.01, 0.02) | 1.01 (0.99, 1.02) | 0.281 |

Note: B = unstandardized regression coefficient; OR = odds ratio; 95% CI = 95% confidence interval; ref = the reference group. The estimates with the *p-values<0.05* are shown in bold

**Table S13**

*Unstandardized Results of the Third Sensitivity Analysis Refitting Models 1-2 (for Alcohol Use Trajectories), Replacing Educational Levels at T_2_ by the One Measured at T_4_ (n = 2,229)*

| **Trajectory** | Model 1 | | | Model 2 | | |
| --- | --- | --- | --- | --- | --- | --- |
| Variable | B (95% CI) | OR (95% CI) | *P-value* | B (95% CI) | OR (95% CI) | *P-value* |
| **Moderate increasing (ref: stable low)** |  |  |  |  |  |  |
| Deprivation | **-0.07 (-0.13, -0.01)** | **0.94 (0.88, 0.99)** | **0.027** | **-0.12 (-0.19, -0.05)** | **0.89 (0.83, 0.95)** | **0.001** |
| **Heavy increasing (ref: stable low)** |  |  |  |  |  |  |
| Deprivation | -0.07 (-0.17, 0.03) | 0.93 (0.84, 1.03) | 0.177 | **-0.15 (-0.26, -0.04)** | **0.86 (0.77, 0.97)** | **0.010** |
| **Early peaking (ref: stable low)** |  |  |  |  |  |  |
| Deprivation | 0.02 (-0.06, 0.09) | 1.02 (0.94, 1.10) | 0.704 | -0.06 (-0.16, 0.04) | 0.94 (-0.86, 1.04) | 0.224 |

Note: B = unstandardized regression coefficient; OR = odds ratio; 95% CI = 95% confidence interval; ref = the reference group. The estimates with the *p-values<0.05* are shown in bold. Only the results for neighborhood socioeconomic deprivation were shown here, which is the only neighborhood-level indicator relevant to this sensitivity analysis

**Fig. S1**

*The Association (Coefficient) between Neighborhood Disorder and Membership in the Moderate-Increasing Alcohol Use Trajectory (vs. the Stable-Low Trajectory) over the Range of values for effortful control in a Johnson-Neyman plot.*


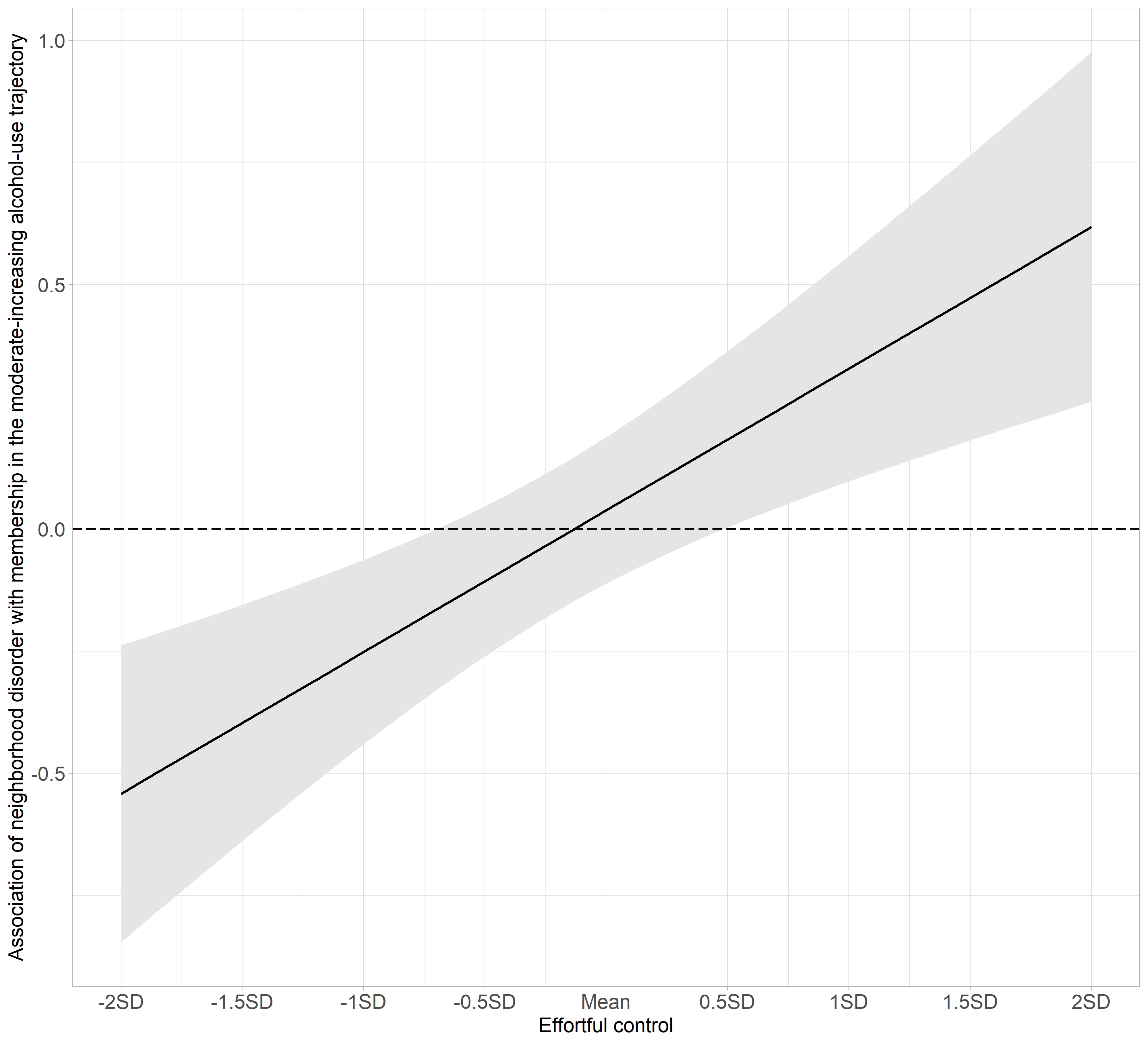


Note: The association was rescaled by the SD of neighborhood disorder. The x-axis indicated the range of effortful control (-2 SD to 2 SD; high values indicate better effortful control). The solid line indicates the varying point estimate of the association at different values of effortful control. The shaded area presents a varying range of upper and lower limits of 95% confidence intervals

**Fig. S2**

*The Association between Neighborhood Social Fragmentation and Membership in the Low Cannabis Use Trajectory (vs. the Never-Use Trajectory) among Adolescents with and without Parental Addiction.*


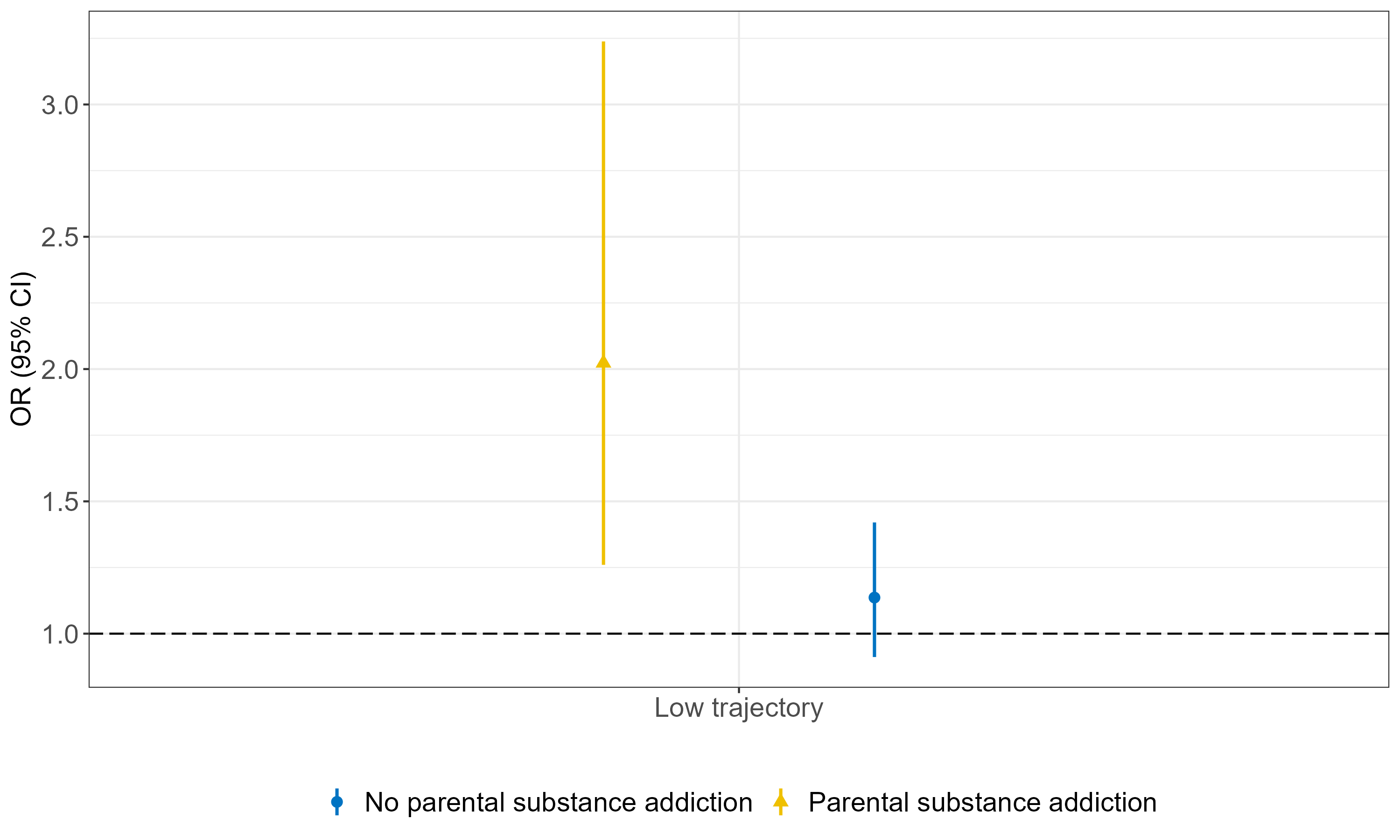


Note: The associations were rescaled by the SD of social fragmentation. OR: odds ratio; 95% CI: 95% confidence interval

**Supplementary materials S1**

*Post-doc analysis 1: Sample Distribution across Combinations of Neighborhood-Level Indicator Quantiles*

The first analysis explored the observation distributions in tabulations to gain further insight into the extent of overlap between neighborhood social fragmentation, socioeconomic deprivation, and disorder (Merlo & Chaix, 2006; Messer et al., 2010; Oakes, 2006). This allowed for evaluating whether the associations estimated in Model 2 (multi-exposure model) were well supported by real data or driven primarily by excessive model-based extrapolation. As presented in the table below, there is a clear indication—consistent with the positive correlations observed—that neighborhoods with higher/lower levels of one indicator tended to be higher/lower in other indicators. About half of the individuals, particularly, fell into cells along the positive diagonal (e.g., from low deprivation-low social fragmentation to high deprivation-high social fragmentation), with almost no observation clustering in cells of the upper-right (e.g., highest deprivation-lowest social fragmentation) and the bottom-left corner (e.g., lowest deprivation-highest social fragmentation). Despite this minor non-positivity issue, the remaining observations were spread over other cells with contrasting levels of different indicators (e.g., 106 adolescents resided in neighborhoods that were highly deprived (3^rd^ quantile) but least low in social fragmentation (1^st^ quantile)).

These results indicated that the observation distributions across the range of combinations of neighborhood-level indicators were not extremely unbalanced. This observed variability ensured that the estimated associations observed in Model 2 were primarily grounded in actual observations, rather than being highly off-support and relying on excessive model-based extrapolations (Merlo & Chaix, 2006; Messer et al., 2010; Oakes, 2006).

*Distribution of Participants and Neighborhoods (in the Bracket) across the Intersection of Quantiles of Neighborhood Socioeconomic Deprivation, Social Fragmentation, and Disorder.*

|  | Quantile of DPI | | | | Quantile of INS | | | |
| --- | --- | --- | --- | --- | --- | --- | --- | --- |
|  | DPI 1 (low) | DPI 2 | DPI 3 | DPI 4 (high) | UNS 1 (low) | UNS 2 | UNS 3 | UNS 4 (high) |
| Quantile of SOF |  |  |  |  |  |  |  |  |
| SOF 1 (low) | 290 (14) | 66 (5) | 106 (9) | 0 (0) | 103 (16) | 339 (8) | 13 (2) | 6 (1) |
| SOF 2 | 186 (9) | 83 (7) | 186 (9) | 44 (3) | 132 (10) | 68 (11) | 214 (6) | 85 (1) |
| SOF 3 | 24 (4) | 151 (8) | 294 (8) | 212 (8) | 2 (2) | 101 (7) | 432 (13) | 145 (5) |
| SOF 4 (high) | 45 (1) | 98 (6) | 32 (4) | 412 (18) | 0 (0) | 14 (1) | 106 (6) | 466 (21) |
| Quantile of DPI |  |  |  |  |  |  |  |  |
| DPI 1 (low) |  |  |  |  | 60 (10) | 343 (12) | 135 (4) | 6 (1) |
| DPI 2 |  |  |  |  | 39 (6) | 91 (8) | 233 (10) | 35 (2) |
| DPI 3 |  |  |  |  | 95 (10) | 18 (4) | 325 (10) | 180 (6) |
| DPI 4 (high) |  |  |  |  | 43 (2) | 70 (3) | 72 (3) | 481 (19) |

Note: DPI, socioeconomic deprivation; INS, disorder; SOF, social fragmentation. Cells are defined as the intersection between quantiles of DPI, INS, or SOF. The number outside the brackets indicates the number of participants; the number inside the brackets indicates the number of neighborhoods

*Post-doc analysis 2: Fitting a Two-Exposure Model by Mutually Adjusting for Social Fragmentation and Socioeconomic Deprivation*

The second analysis fitted a two-exposure model to include social fragmentation and socioeconomic deprivation (excluding disorder) simultaneously. This analysis aimed to determine whether the three associations observed exclusively in Model 2 (multi-exposure model) remained significant in the simpler two-exposure model, while also evaluating whether these results demonstrated greater precision, reflected by narrower confidence intervals (or smaller standard errors) compared to those in Model 2 (multi-exposure model). Here, the focus was on the three associations that were significant exclusively in Model 2, but not in Model 1, from the main analyses: 1) higher social fragmentation was associated with higher odds of being in the early-peaking alcohol use trajectory (vs. the stable-low trajectory); 2) higher social fragmentation was associated with higher odds of being in the heavy-increasing alcohol use trajectory (vs. the stable-low trajectory); 3) higher socioeconomic deprivation was associated with lower odds of being in the heavy-increasing alcohol use trajectory (vs. the stable-low trajectory). As shown in the table below, all three associations that were exclusively significant in Model 2 but not in Model 1 remained significant in the two-exposure model. Specifically, the point estimates for these associations in the two-exposure model were similar to those in Model 2 (multi-exposure model), while their standard errors were notably smaller, indicating greater precision. These results indicated the robustness of the three associations observed in Model 2 from the main analyses, despite the relatively wider confidence intervals of these associations in that model.

*Unstandardized Associations of Social Fragmentation and Socioeconomic Deprivation with Trajectories of Alcohol Use in the Single-, Two-, and Multiple-Exposure Models*

| **Trajectory** | Single-exposure model (Model 1) | Two-exposure model (social fragmentation + socioeconomic deprivation) | Multi-exposure model (Model 2; social fragmentation + socioeconomic deprivation + disorder) |
| --- | --- | --- | --- |
| Variable | OR (95% CI); SE | OR (95% CI); SE | OR (95% CI); SE |
| **Moderate increasing** |  |  |  |
| Social fragmentation | 1.00 (0.95, 1.06); 0.028 | 1.07 (1.00, 1.14); 0.032 | 1.06 (0.98, 1.14); 0.036 |
| Socioeconomic deprivation | 0.94 (0.88, 0.99); 0.030 | 0.90 (0.84, 0.96); 0.033 | 0.89 (0.83, 0.95); 0.035 |
| **Heavy increasing** |  |  |  |
| Social fragmentation | 1.04 (0.96, 1.12); 0.039 | 1.13 (1.05, 1.23); 0.041 | 1.16 (1.06, 1.28); 0.049 |
| Socioeconomic deprivation | 0.93 (0.84, 1.03); 0.051 | 0.86 (0.77, 0.95); 0.051 | 0.87 (0.78, 0.97); 0.058 |
| **Early peaking** |  |  |  |
| Social fragmentation | 1.07 (0.99, 1.15); 0.037 | 1.11 (1.01, 1.22); 0.049 | 1.11 (1.00, 1.22); 0.051 |
| Socioeconomic deprivation | 1.02 (0.94, 1.10); 0.039 | 0.94 (0.86, 1.04); 0.049 | 0.94 (0.85, 1.04); 0.050 |

Note: OR = odds ratio; SE = standard error

**Reference**

Merlo, J., & Chaix, B. (2006). Neighbourhood effects and the real world beyond randomized community trials: a reply to Michael J Oakes. *International Journal of Epidemiology*, *35*(5), 1361-1363. <https://doi.org/10.1093/ije/dyl211>

Messer, L. C., Oakes, J. M., & Mason, S. (2010). Effects of Socioeconomic and Racial Residential Segregation on Preterm Birth: A Cautionary Tale of Structural Confounding. *American Journal of Epidemiology*, *171*(6), 664-673. <https://doi.org/10.1093/aje/kwp435>

Oakes, J. M. (2006). Commentary: Advancing neighbourhood-effects research—selection, inferential support, and structural confounding. *International Journal of Epidemiology*, *35*(3), 643-647. <https://doi.org/10.1093/ije/dyl054>
